# Exploring the impacts of Covid-19 on the electronic product trade of the G-7 countries: A complex network analysis approach and panel data analysis

**DOI:** 10.1371/journal.pone.0286694

**Published:** 2023-09-29

**Authors:** Halil Özekicioğlu, Burcu Yilmaz, Gamze Alkan, Suzan Oğuz, Ceren Kocabaş, Fatih Boz

**Affiliations:** 1 Department of International Trade and Logistics, Faculty of Applied Sciences, Akdeniz University, Konyaaltı, Antalya, Türkiye; 2 Department of International Trade and Logistics, Institute of Social Sciences, Akdeniz University, Konyaaltı, Antalya, Türkiye; 3 Independent Researcher, Antalya, Türkiye; 4 Independent Researcher, Mersin, Türkiye; 5 Department of Foreign Trade, Finike Vocational School, Akdeniz University, Finike, Antalya, Türkiye; Northwest Agriculture and Forestry University China, CHINA

## Abstract

The present study attempts to explore the impacts of COVID-19 on the intra-group electronic product trade of the world’s seven largest economies. In line with this purpose, we performed a complex network analysis of the electronic product trade of the group of seven (G-7) countries and China, as well as a panel data study comprising solely the G-7 countries. In this regard, we investigated the trade networks within the G-7 countries, to which China has been added, and determined the prominent countries in the network during the pandemic to be China, the USA and Canada. The findings also revealed that China, one of the pioneering countries in electronic product trade, has the most ties in electronic products exports with the USA, the other countries with which the USA had the most ties were Japan and Germany, apart from Canada. It was discovered that Germany was the most active country in the network, following the USA, in terms of export ties and the number of export countries in its network. The panel data analysis, on the other hand, yielded two different models, namely import and export, based on 22 months of data, from March 2020 to December 2021, considering the World Health Organization’s (WHO) declaration of COVID-19 as a pandemic on March 11, 2020. The findings showed that independent variables affecting the electronic product trade within G-7 countries bore different effects in both models, that the deaths/cases ratio, the tests/cases ratio and the number of cases had adverse impacts while the population had positive impacts on exports in the first model, and that the tests/population ratio had adverse effects while the number of tests and the population had positive impacts on intra-group electronic product imports.

## 1. Introduction

It is evident that the electronics industry has grown exponentially thanks to technological advancements. Countries that adapt quickly to changes and transformations and prioritize their policies on digitalization have caught elusive momentum in development in a short time. In this regard, it is prudent to assert that countries desiring to grow in the electronics industry prioritize policies on investments in research and development (R&D). The increased trade volume in this industry also contributes to the acceleration of technological advancements. Such advancements then globalize trade, help increase countries’ trade shares and competitiveness and decrease production costs, and provide consumers with convenient consumption opportunities at much more affordable prices.

Online shopping and electronic goods trades are closely linked. The development of E-commerce has resulted in the growth of the electronic product sector. The share of electronic products in e-commerce sales is at the top of the e-commerce wars. One of the sectors that benefited the most from the Covid-19 pandemic is the electronic products sector [1]. In addition, the growth of the electronic products sector is directly related to rising income levels. Although the decline in income levels in shocking crises, such as the Covid-19 pandemic negatively affected sales to some extent, the lengthy times spent at home attracted people to internet shopping, which led to an increase in electronic product sales. The Covid-19 pandemic caused individuals to spend more time in front of computers for shopping, and sales of electronic products increased during the pandemic [2].

The demand for electronic products has experienced an exceptional surge in recent times, particularly since the beginning of the 21st century, with technological advancements playing a crucial role in fueling this trend. Considering the world electronics industry, electronics imports and exports have increased by 219% and 210%, respectively, in the last 20 years. It can be argued that this increase is due to a reciprocal contribution between technological developments and the internet. In other words, the proliferation of the internet since 1995 has accelerated technological progress, and technological advancements have in turn expanded the use of the internet. It should be noted that technological advancements with the spread of the internet in 1995 brought great impacts on these increases. In this 20-year period, the leading importing and exporting countries in electronics have become Viet Nam, China, and Hong Kong. Such an increase has been led by the Asian region’s becoming a global electronics production center thanks to their export-oriented industrialization strategies and R&D investments. For example, China has rapidly developed and increased its production capacity since the 2000s, thus becoming the global center of production chains. Vietnam’s electronics industry has grown significantly in less than a decade, which has been influenced by Vietnam’s participation in the World Trade Organization (WTO), the liberalization of its trade through an agreement with the USA, an attracting investment environment in the country, and its geographical proximity to electronic parts and components suppliers such as China, as well as its low-cost workforce [3]. In addition, it has been noted that natural, economic, and technological factors, as well as international and national policies and regional cultures, play a crucial role in the development of global electronic product trade [4]. For example, China’s geographic and political advantages, as well as the Belt and Road Initiative’s efforts to increase commercial activity and regional cooperation among countries along its route, improve infrastructure, and encourage investment to strengthen connectivity among these countries, are factors that have influenced China’s significant position in global electronic product trade [4].

Leading countries in global electronic product trade stand out not only for their population and technological advancements but also for their competitiveness. Given the global electronic product trade, it is noteworthy that three of the top 10 countries are G-7 members (the USA, Germany, and Japan) while China exhibits a noteworthy presence in the upper echelons of this classification, outside the G-7. According to the Competitive Industrial Performance (CIP) Index, these four countries are among the top four most competitive economies across the world [5]. Therefore, they, together with Asian countries, have an influential say in the electronics trade in the world. The share of the G-7 countries in total world trade is 37% in imports and 34% in exports. In addition, these countries realize 22% of the world’s high-tech product exports (26% of the imports and 19% of the exports). The trade statistics of these countries have hit rates mentioned from 40% in the last 20 years due to the severe impacts of the pandemic, as well as the shift of production centers to countries in the Asia-Pacific Region for seeking cheap labor and low-cost production and, therefore, increasing the share of East and Southeast Asian countries in the world electronics trade. Given the world’s electronics exports, China, South Korea, and Singapore, as the newly industrialized countries in the last 20 years, have acquired their seats as influential countries along with the industrialized countries.

The global trade volume, and hence the electronic product sector, is deeply affected by exceptional circumstances such as pandemics, earthquakes, wars, and natural disasters that occur worldwide [6–9]. COVID-19, coming out at the end of 2019 and affecting the whole world as of 2020, quickly spread to many regions and countries, exacerbating the number of cases worldwide. The increase in the number of cases and deaths mandated many countries to apply various restrictions and quarantine measures to decelerate the spread of the pandemic. Social and economic activities were restricted in such a setting where a global health crisis was at the gate, severely affecting individual and business mobility. The Covid-19 pandemic’s most significant economic consequence has been the considerable reduction in household and business incomes, resulting in financial risk that has disrupted the normal functioning of households, firms, the financial sector, and the public sector. Given the highly evolved interconnectivity between sectors, an increase in financial risk in one sector has the potential to spread to others [10]. Although the impact of Covid-19 on the economy has been widespread, certain sectors have experienced an accelerating effect from the crisis, notably in areas such as online commerce, distance learning, telemedicine, and geopolitics, as well as in advanced electronic products, high technology, and medical technology fields, where it has acted as a driving force for progress [1].

While the G-7 countries’ electronics exports were 20% just before the pandemic, they became 18% during the pandemic. The same shrinkage happened at 3% in the industry worldwide due to the impacts of the pandemic and the disruptions in the supply chain. However, a 5–7% regional growth is expected in the industry for 2022, thanks to decreased pandemic-related impacts worldwide in 2021 [11]. Owing to restrictions during the Covid-19 period, many companies have transitioned to remote working systems, and distance education has become widespread, leading to an increase in demand for the equipment required for these activities. The World Trade Organization (WTO) stated that the trade of computer and electronic components required for distance work grew by 4% and 12%, respectively, after the first quarter of 2020, and this growth increased to 28% in the first quarter of 2021 [12].

The rapid spread of the virus brought social, economic, and commercial relations to a standstill on a global scale. The fact that China, hosting noteworthy production centers, was the center of the outbreak also damaged the functioning of the global supply chain and disrupted the flow of goods and services between countries. Hence, this study employed network analysis to assess the impact of trade between the world’s seven largest economies and China as eighth economy on the electronic product sector, which holds a substantial share in international trade.

As previously mentioned, it is crucial to comprehend the role of G-7 and China in electronic product trade and determine how this trade has evolved and been affected during the pandemic. The global electronic product trade network is a complex structure because of the increased commercial connections between countries engaged in electronic product trade, and the close trade relations between countries further increase the network density [4].

The literature reveals that studies on the complex network analysis of trade have been conducted in different fields, with different perspectives and within different contexts, covering various products, countries, and economic formations. Studies utilizing network analysis have been conducted on global trade contexts [13–18], intra-African countries [19], ASEAN countries [15, 20], OECD countries [21], China, BRIC, and African countries [22, 23], Latin American countries [24], Shanghai Cooperation Organization countries [25], and Central Asia and Caucasus countries [26]. Furthermore, these studies focus on general trade [15, 17–19, 21, 22, 24–27], energy [13, 14, 16, 23, 28], food [29–31], clothing [32, 33], and electronic products [4]. However, to the best of our knowledge, no studies have examined electronic product trade at the country group level during the COVID-19 period using complex network and panel data analyses. Accordingly, this study expounded on the extent to which the electronics industry was affected by the pandemic on the basis of statistical data. Moreover, we visualized the positions of the China and G-7 countries in the intra-group electronics trade network, determined the hub and authority countries in the network, and discussed the changes in the trade network. In addition, panel data analysis was employed to examine the impact of Covid-19 on the electronic product trade of G-7 countries.

## 2. Electronics trade in the world and the G-7 countries

The electronics industry, the popularity of which has gradually increased thanks to the widespread use of the internet since 1995, mainly targets introducing various equipment, parts, and devices to accelerate and ensure the continuity of technological advancements. Even the modern tech world has already adapted to automation in industrial production and the “Internet of Things”. In the world, the major markets for electronics trade are China, the USA, Japan, South Korea, and Germany [11]. The electronic product industry is among the most profitable sectors, thanks to the increase in demand and prices, even during the period when the effects of the pandemic persisted. Semiconductor products in this industry have been the pioneer of this immense market. The fact that global sales reached an all-time high of $553 billion in 2021 and showed an extraordinary annual increase of 26% clearly demonstrates the demand for this industry. However, it is expected that the final demand growth in this industry will normalize, and new production capacities will increase starting in 2022 [34].

**Fig 1:**
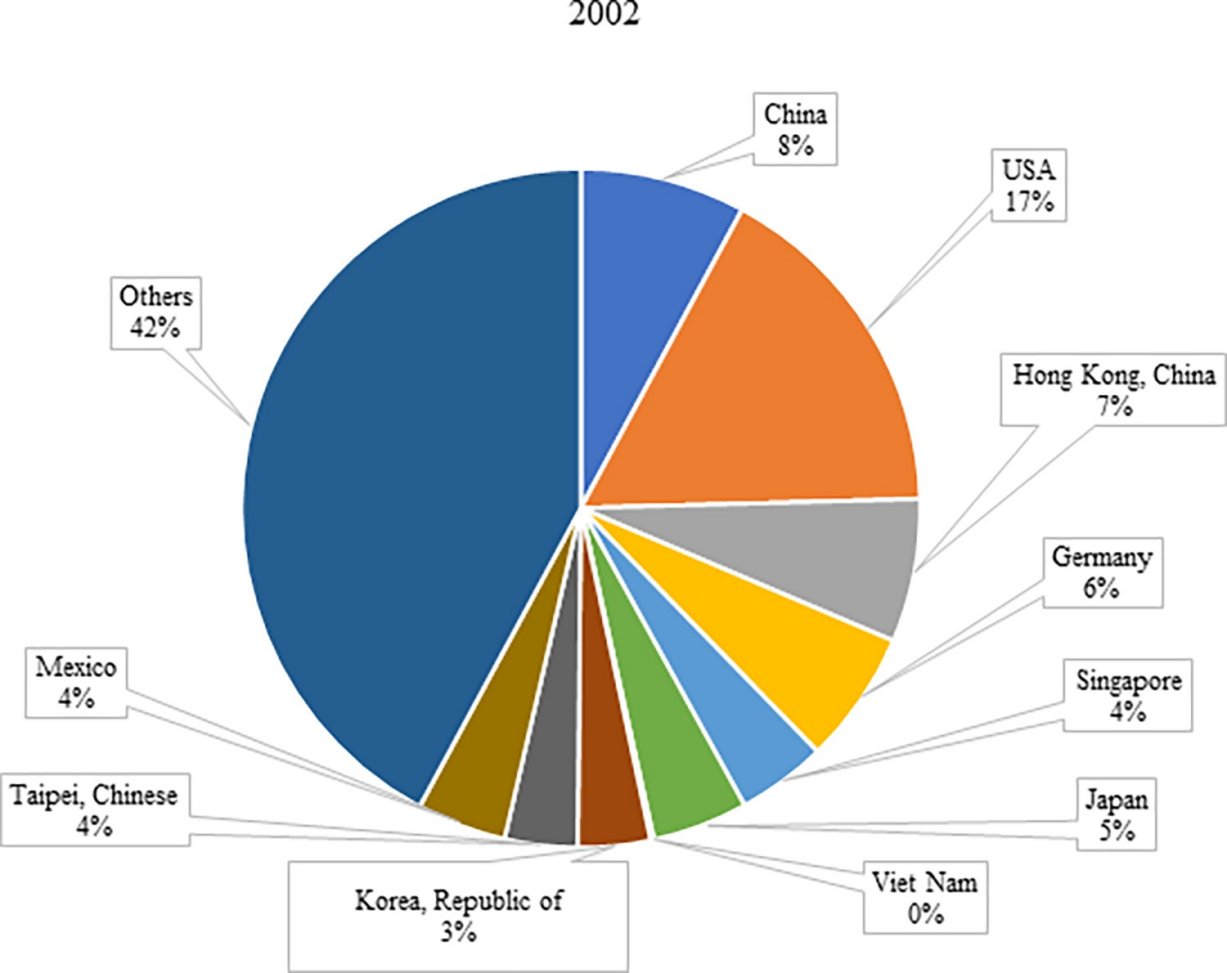
Share of countries in the top 10 in 2020 in world electronic product imports in 2002. **(Adopted from:** Trademap 2022**)**.

**Fig 2:**
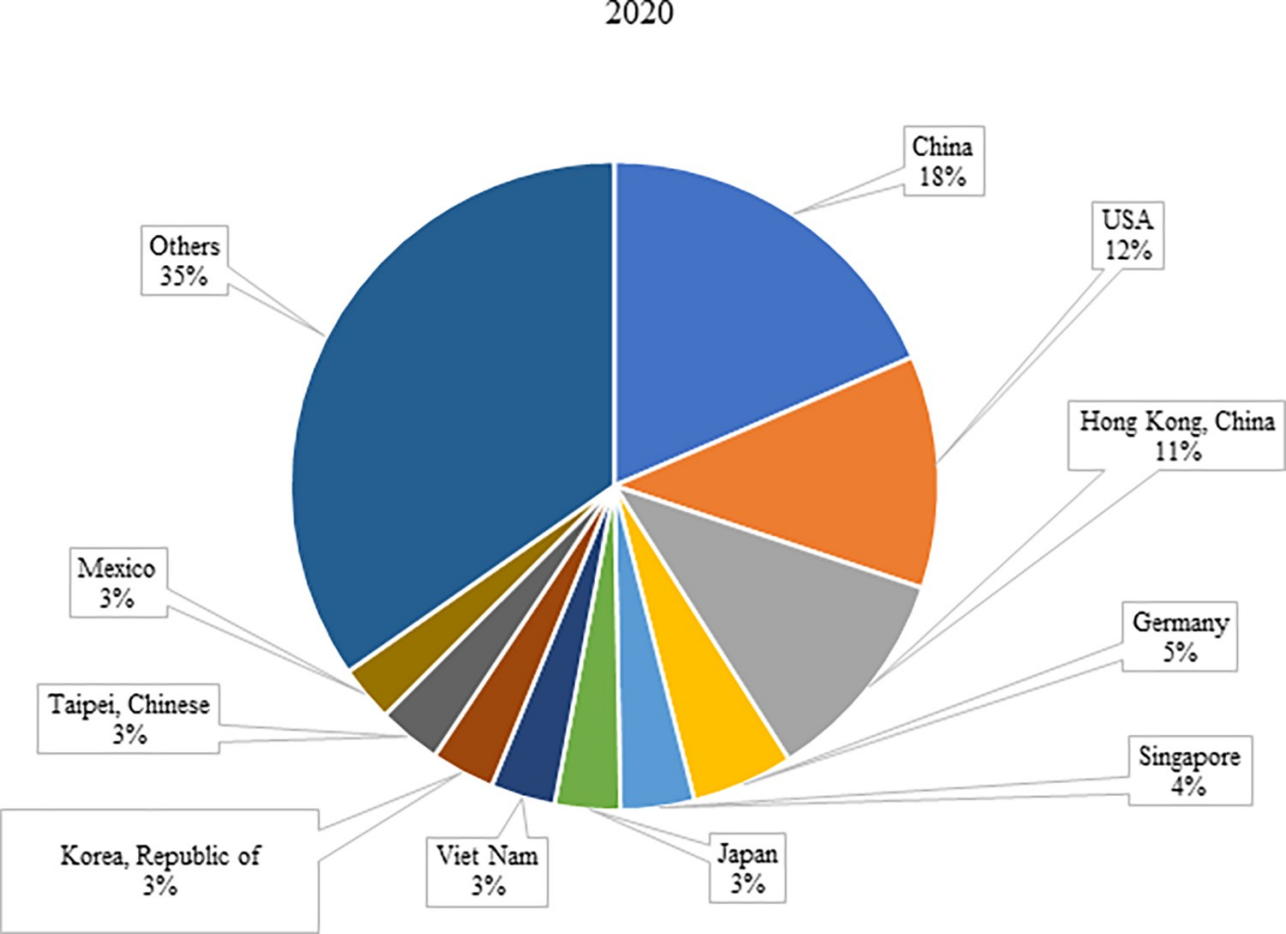
Share of top 10 countries in world electronic product imports in 2020. **(Adopted from:** Trademap 2022**)**.

The analysis of global electronic product trade was conducted using the Trademap dataset [35]. The United Nations’ Classifications on Economic Statistics [36] defines the category of electronic products with the Code 85 as "Electrical machinery and equipment and parts thereof; sound recorders and reproducers; television image and sound recorders and reproducers, parts and accessories of such articles." Therefore, this study focuses on electronic product trade under Code 85 in the Trademap dataset. In this context, According to Table 1, global electronic product imports have been increasing over the years. The global electronic imports have increased by about 219%, reaching $2.96 trillion in 2020 from $929 billion in 2002, largely due to technological advancements in the industry over the past two decades. Accordingly, the countries with the highest increase in imports have become Viet Nam (6,630%), China (649%), and Hong Kong (408%), respectively. The share of these countries in global electronic product imports has increased significantly from 0.15%, 7.89%, and 6.82% in 2002 to 3.22%, 18.51%, and 10.86% in 2020, respectively (Figs 1 and 2). The economic reforms in Vietnam have uplifted this country from the league of lowest-income nations. The government has supported the establishment of private enterprises and attempted to revive international trade by reducing tariff and non-tariff barriers on imports and exports. Moreover, the country’s accession to the WTO in 2007 has further accelerated the flow of international trade, leading to robust export growth [37]. Hong Kong has also gained an important seat in world trade thanks to the following reforms: effective and consistent public and fiscal policies, encouraging low tax rates and foreign direct investments, ensuring stability in the exchange rate between Hong Kong Dollar and US Dollar, promoting confidence in Hong Kong Dollar and expanding trade with it as a legal payment tool, increasing the production capacity as a result of public infrastructure investments and imports of large capital goods, and encouraging people living abroad to return to Hong Kong and join the workforce [38].

**Table 1:** World electronic products imports (million $) and increase rates between 2002–2020 (%).

Countries	2002	2015	2016	2017	2018	2019	2020	2002–2020 Increase Rate (%)
China	73,245	431,611	414,338	455,495	521,542	497,420	548,420	649
USA	155,001	333,456	335,583	355,993	366,232	351,074	343,401	122
Hong Kong, China	63,339	266,139	276,734	303,438	327,906	307,677	321,671	408
Germany	58,837	124,423	130,625	145,974	159,503	151,587	150,246	155
Singapore	39,575	84,870	84,915	93,058	101,724	98,127	108,751	175
Japan	42,397	90,196	90,004	97,744	101,197	98,779	96,698	128
Viet Nam	1,418	41,857	47,732	63,777	67,899	77,772	95,444	6,630
Korea, Republic of	32,002	77,756	75,145	82,864	84,757	89,696	94,107	194
Taipei, Chinese	32,222	53,306	58,336	67,336	75,376	79,599	93,178	189
Mexico	39,646	85,410	84,243	85,866	94,899	94,771	82,867	109
Others	391,175	883,864	873,380	995,211	1,074,557	1,062,955	1,027,783	163
**Total**	**928,857**	**2,469,379**	**2,467,802**	**2,745,840**	**2,974,278**	**2,903,507**	**2,962,193**	**219**

**Adopted from:** Trademap 2022

**Fig 3:**
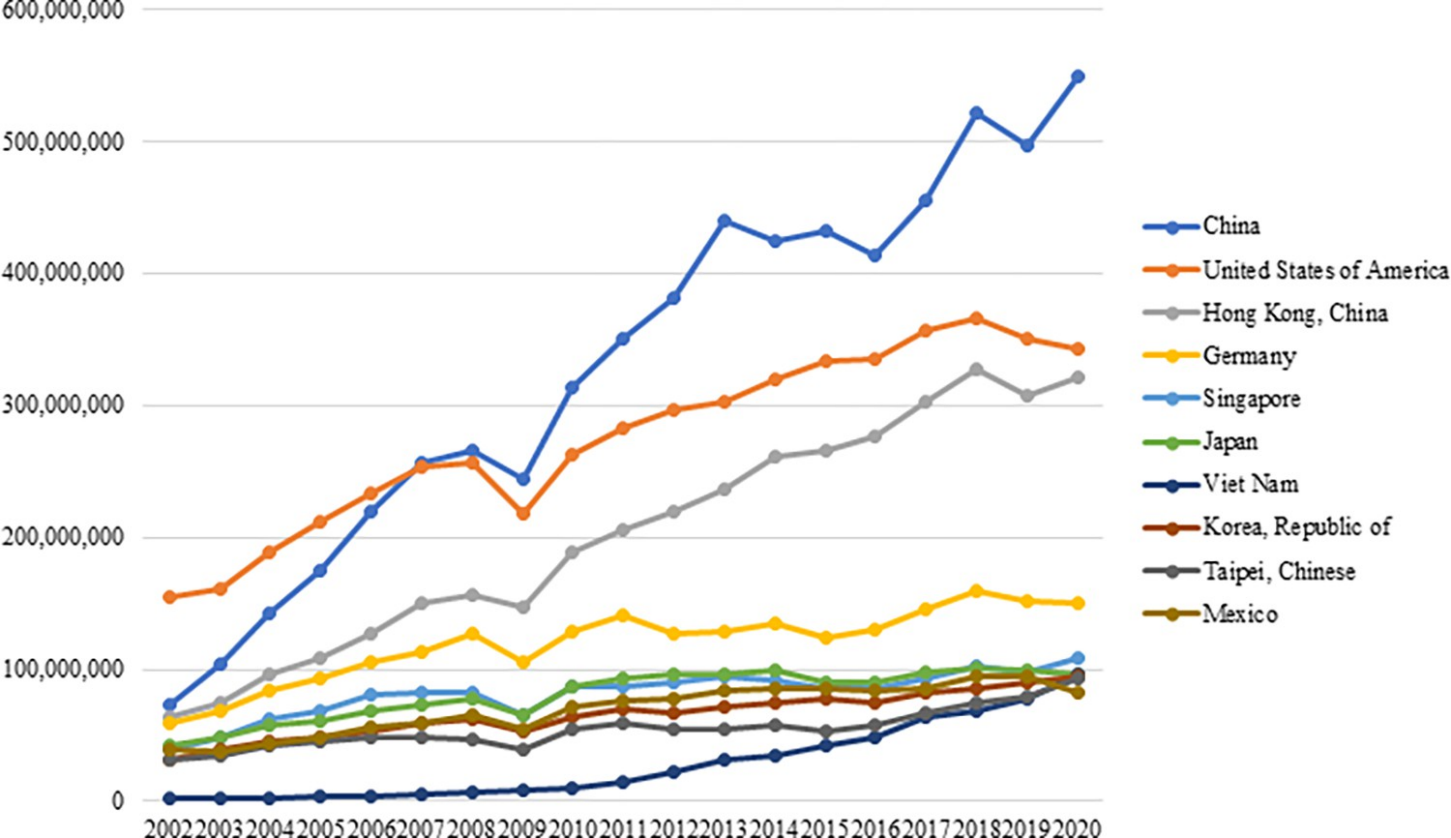
Top 10 countries with the highest imports of electronic products in the world between 2002–2020 (thousand $). **(Adopted from:** Trademap 2022**)**.

The countries importing the most electronic products worldwide became China, the USA, Hong Kong, Germany, Singapore, and Japan in 2020 (Fig 3). In the same year, China ranked first in world imports with an import volume of approximately $549 billion, followed by the USA with an import volume of $343 billion.

**Fig 4:**
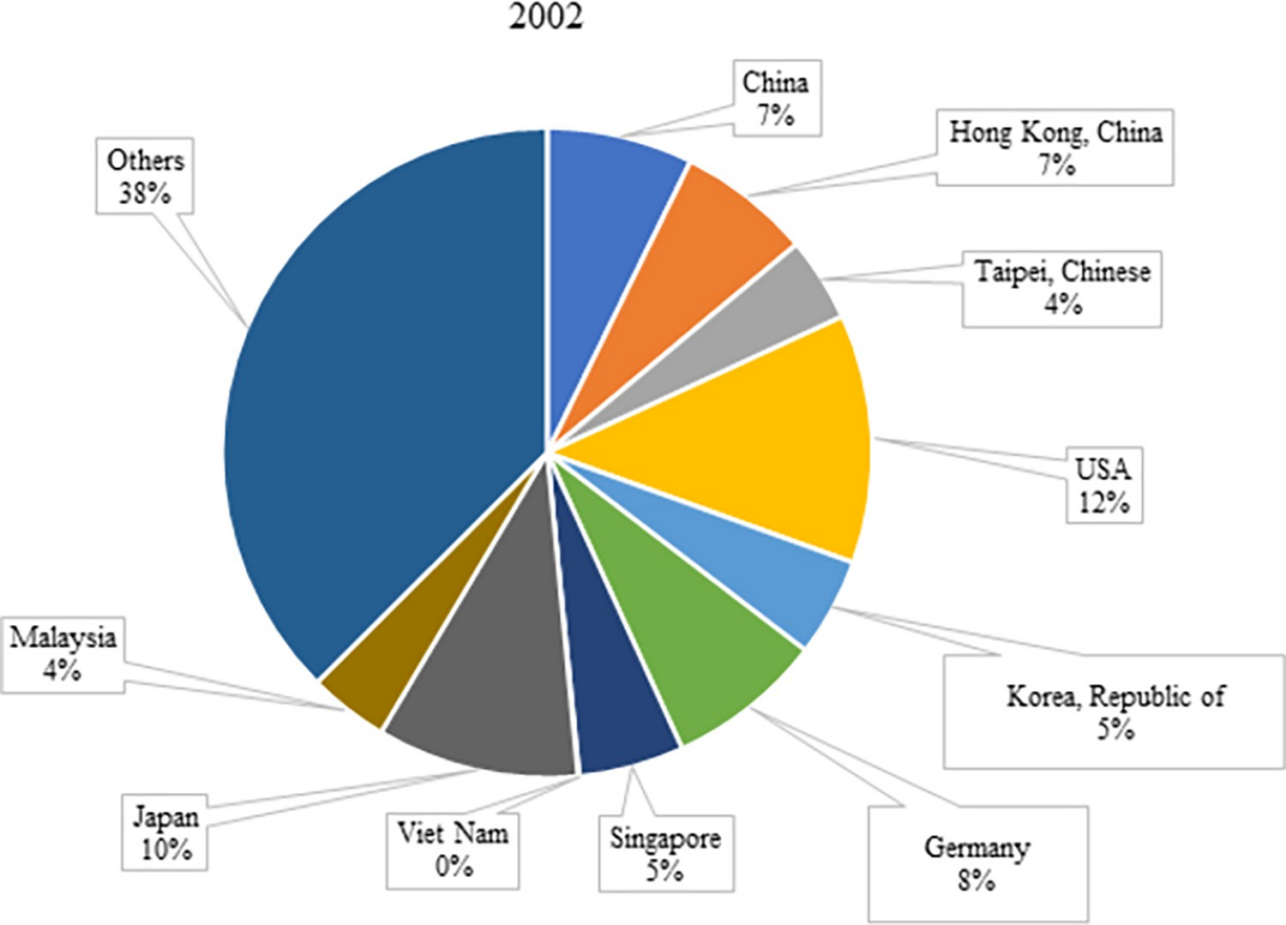
Share of countries in the top 10 in 2020 in world electronic product exports in 2002. **(Adopted from:** Trademap 2022**)**.

**Fig 5:**
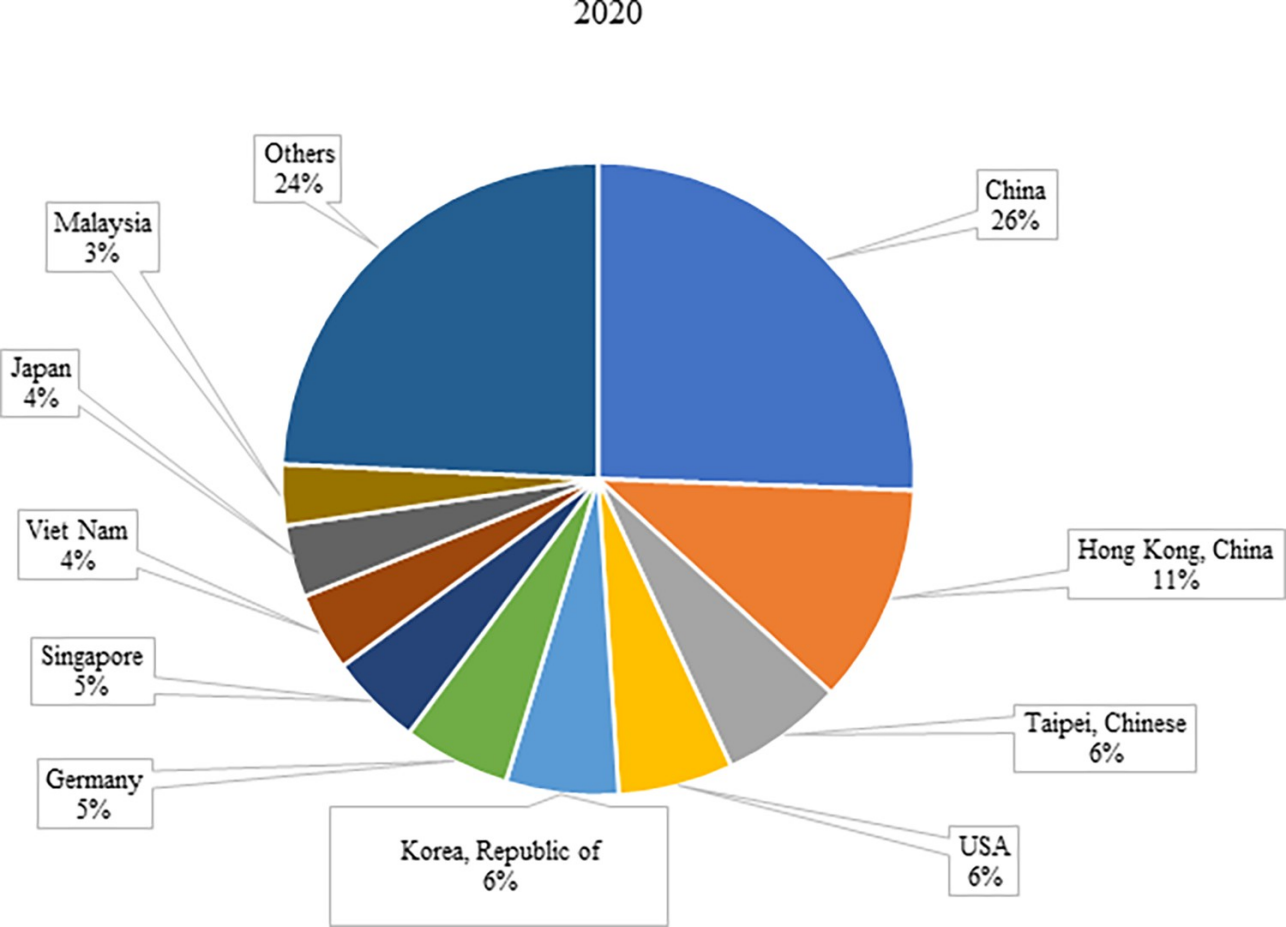
Share of top 10 countries in world electronic product exports in 2020. **(Adopted from:** Trademap 2022**)**.

Global electronic product exports have been increasing in the last 20 years; the world has witnessed an increase of about 210% in electronic products exports over the years. According to Table 2, the countries with the highest increase in exports in the last 20-year period have become Viet Nam (16,241%), China (990%), and Hong Kong (425%), respectively. The share of these countries in global electronic product exports increased from 0.08%, 7.28%, and 6.63% in 2002 to 4.00%, 25.58%, and 11.23% in 2020, respectively (Figs 4 and 5). The share of electronics trade in China’s total trade has increased significantly since the 1990s. While manufacturing products such as refrigerators and washing machines in the 1990s, it has shone out in the production and exports of high-tech products such as computers and mobile phones since the 2000s [39]. Vietnam’s electronics, computers, and components exports also grew at an annual average rate of 28.6%, even between 2010–2020, the period before the US-China trade wars and COVID-19 quarantine practices. It can be asserted that Investment Law and Enterprises Law entered into force in 2000 and 2005, respectively, in Vietnam contributed to this growth [40].

**Table 2:** World electronic products exports (million $) and increase rates between 2002–2020 (%).

Countries	2002	2015	2016	2017	2018	2019	2020	2002–2020 Increase Rate (%)
China	65,114	600,292	557,062	598,975	664,425	670,448	709,933	990
Hong Kong, China	59,307	249,295	259,511	281,509	309,722	300,863	311,586	425
Taipei, Chinese	37,531	116,099	123,907	141,381	144,380	147,394	174,276	364
USA	110,607	170,011	166,964	174,505	176,508	173,001	162,785	47
Korea, Republic of	43,928	138,365	134,303	163,093	184,609	153,643	159,681	264
Germany	69,640	130,043	136,073	149,505	163,965	158,451	152,034	118
Singapore	46,831	118,214	114,845	124,067	128,889	120,691	131,983	182
Viet Nam	680	47,400	57,193	75,323	86,600	97,158	111,093	16,241
Japan	89,793	95,621	98,251	105,537	109,423	103,041	102,497	14
Malaysia	35,598	59,845	58,417	68,709	83,104	81,965	86,404	143
Others	335,164	580,634	578,090	642,442	695,946	695,649	672,855	101
**Total**	**894,193**	**2,305,819**	**2,284,616**	**2,525,047**	**2,747,571**	**2,702,304**	**2,775,125**	**210**

**Adopted from:** Trademap 2022

**Fig 6:**
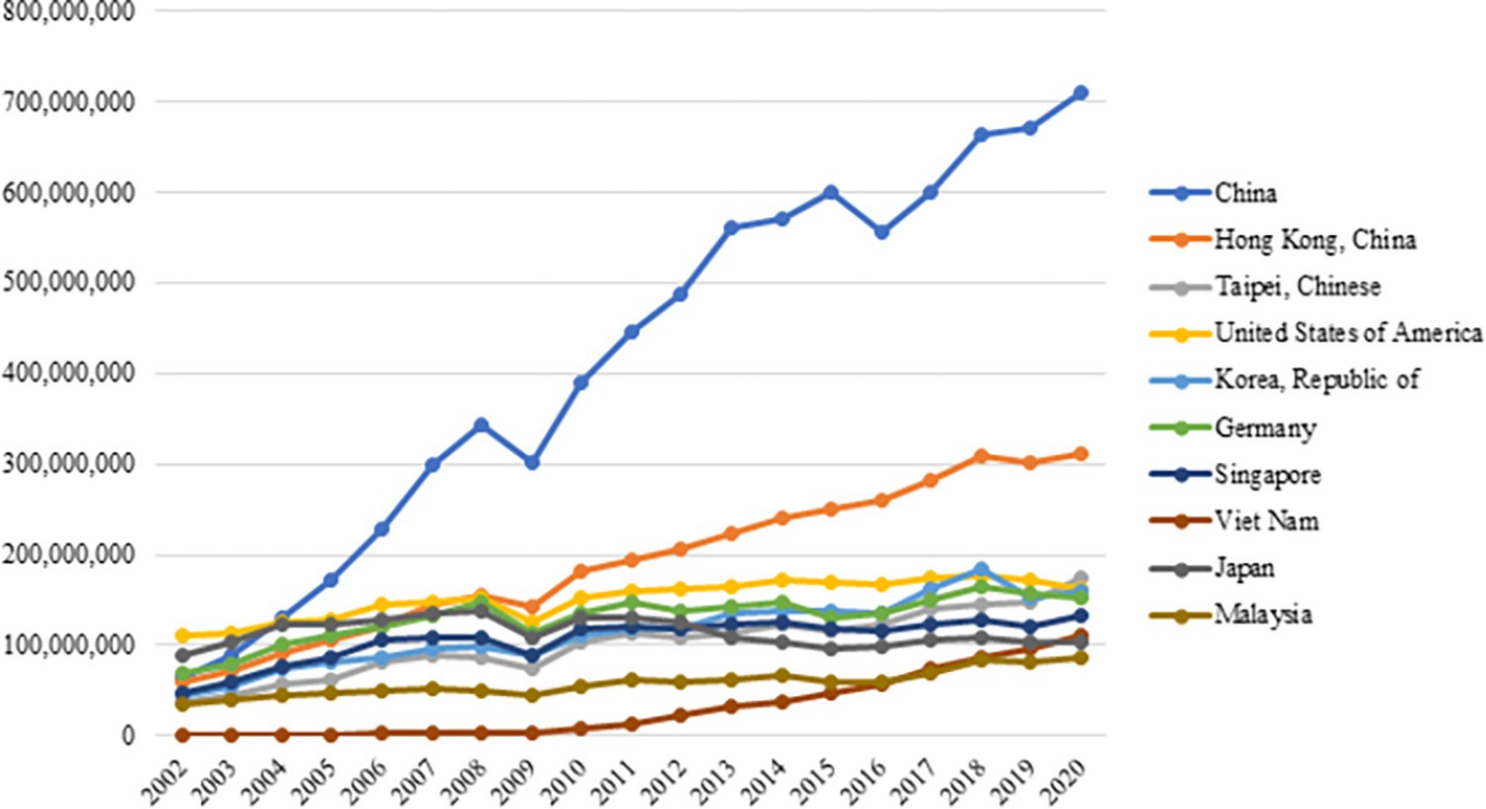
Top 10 countries exporting the most electronic products in the world between 2002–2020. **(Adopted from:** Trademap 2022**)**.

Fig 6 demonstrates the top 10 countries exporting the most electronic products worldwide. In 2020, China ranked first in electronics exports with an export volume of 710 billion dollars, followed by Hong Kong with an export volume of $312 billion. The USA ranked fourth with an export volume of $163 billion. According to the figure, China is the leader compared to other countries, while South Korea, Germany, Singapore, and Japan are among the leading countries in world electronics exports.

**Fig 7:**
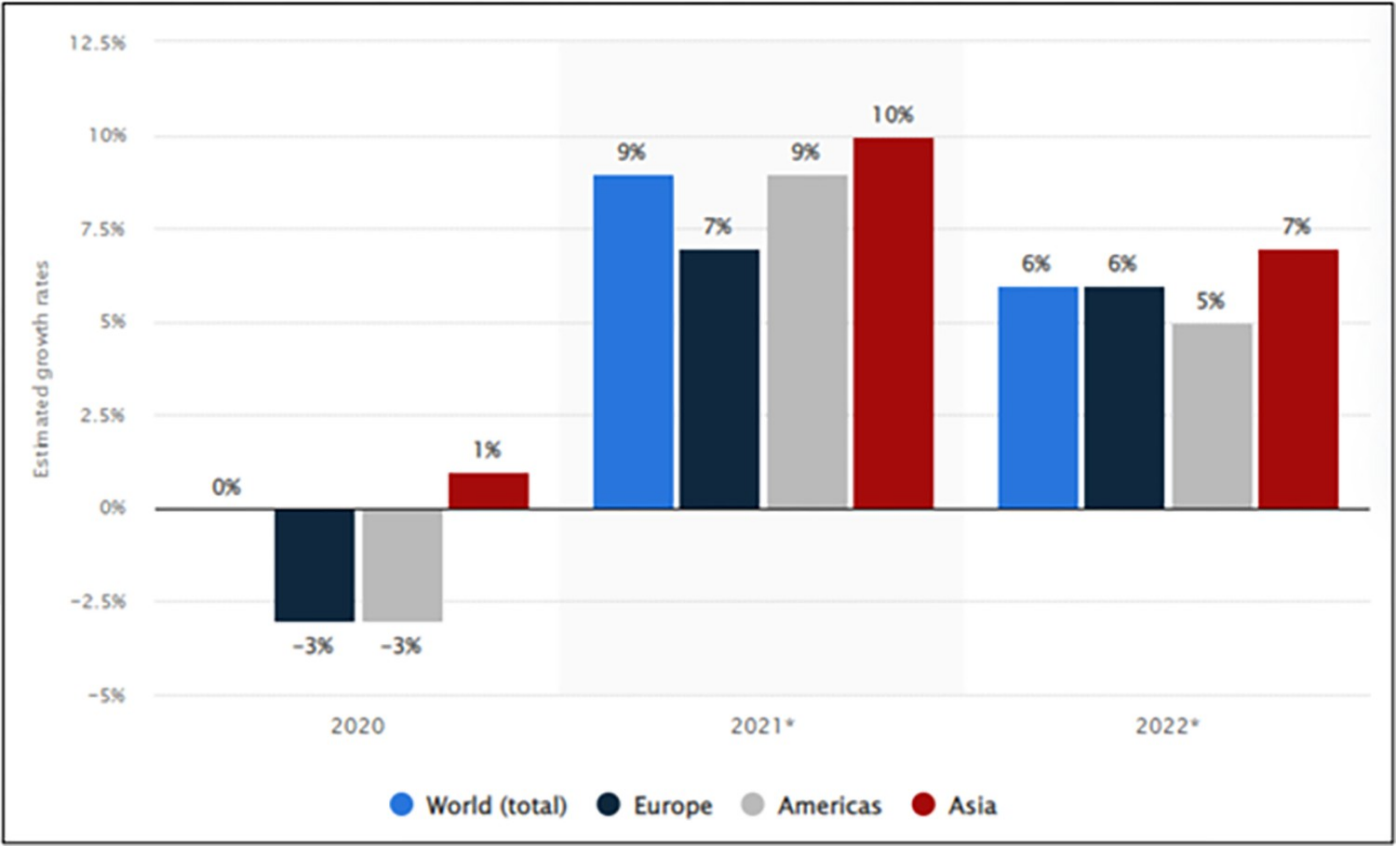
Estimated growth rates for the global electronics industry in 2020, 2021, and 2022. **(Adopted from:** Statista 2022**)**.

Fig 7 shows the estimated growth rates of the global electronics industry by region between 2020–2022. The Asian Region ranked first with a growth of about 10% in 2021. In 2022, the electronics industry in the United States is expected to grow by around 5% annually. In 2020, there was a 3% contraction in the market due to the impacts of the pandemic and the disruptions in the supply chain. In 2022, the electronic product market is predicted to grow and overcome the effects of the pandemic.

The G-7 countries, around which a significant part of the global economy and trade revolves, is an intergovernmental political forum consisting of Germany, the USA, France, Italy, Canada, and Japan. In particular, the USA, Japan, and Germany are the leading countries in the world electronics trade. While the share of G-7 nations in world electronics imports was around 40% in 2002, it dropped to 26% in 2020 [35].

**Fig 8:**
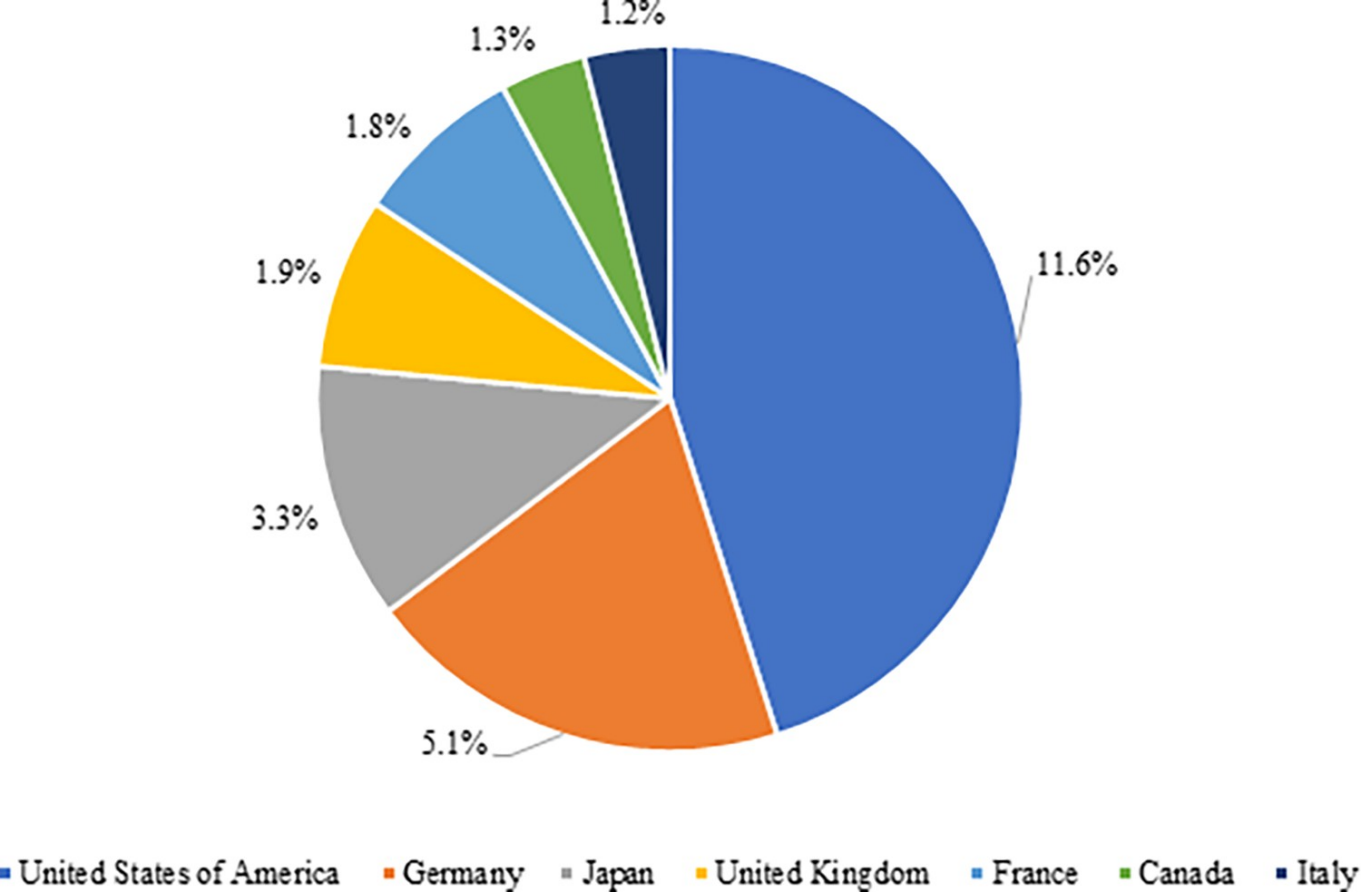
Share of G-7 countries in world electronic product imports in 2020. **(Adopted from:** Trademap 2022**)**.

This decline is most probably because of due to the severe impacts of the pandemic, as well as the shift of production centers to countries in the Asia-Pacific Region for seeking cheap labor and low-cost production and, therefore, increasing the share of East and Southeast Asian countries in the world electronics trade. As in Fig 8, the USA (11.6%), Germany (5.1%), and Japan (3.3%) were those with the highest share of world electronics imports within the G-7 countries in 2020.

**Fig 9:**
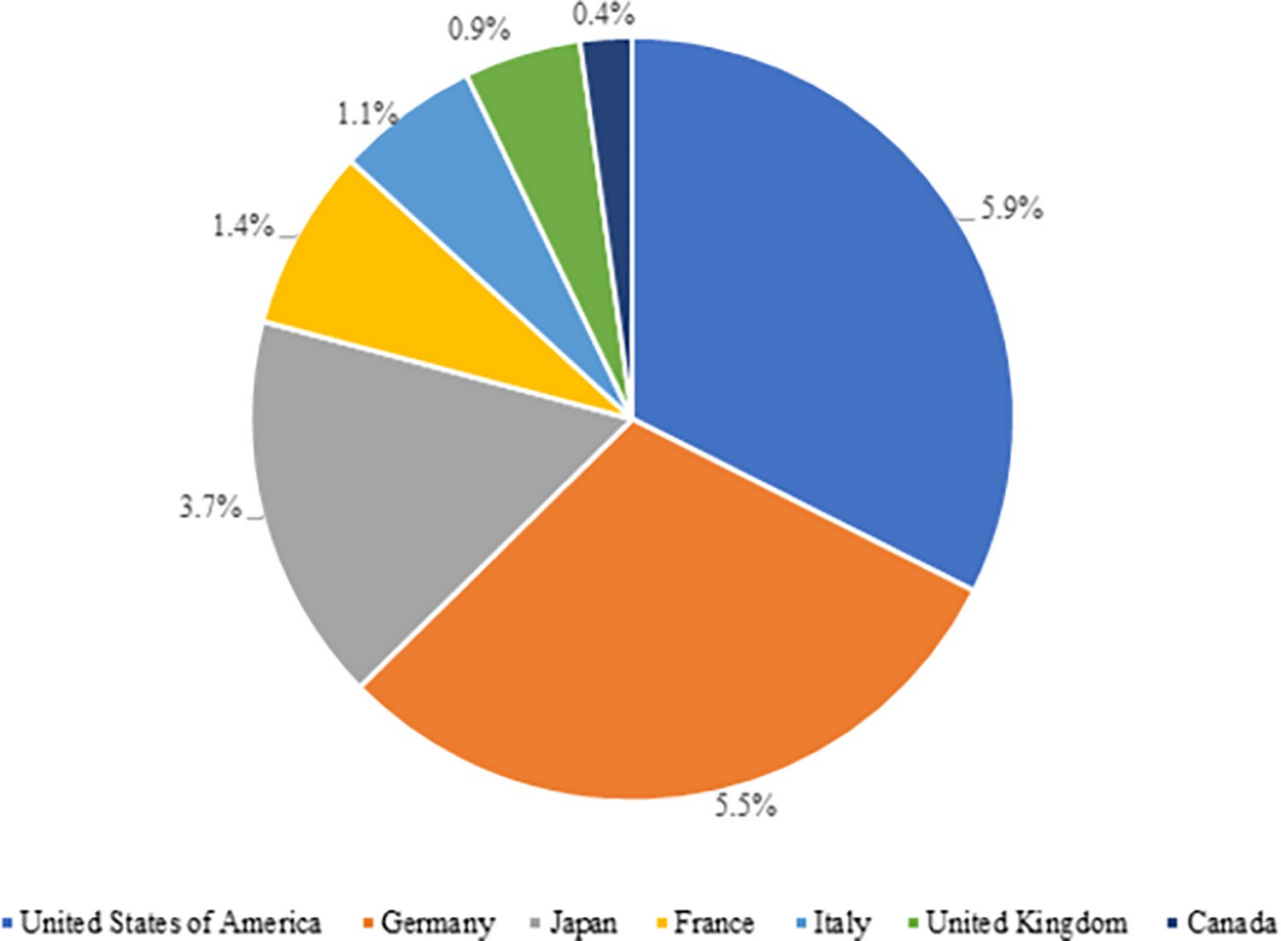
Share of G-7 countries in world electronic product exports in 2020. **(Adopted from:** Trademap 2022**)**.

The global electronics exports of the G-7 countries have been declining over the years. While the share of these countries in world electronics exports hit 42% in 2002, it dropped to 19% in 2020 [35]. This drop may be accounted for by China’s increasing import rates with the impacts of its industrial policies for the last 20 years and the effects of the pandemic. As in Fig 9, the USA (5.9%), Germany (5.5%), and Japan (3.7%) became those with the highest share in global electronics exports within the G-7 countries in 2020.

The shares of the G-7 countries in world electronics exports are shown in Table 3. According to the 2019 Trademap data, the G-7 countries realized 20.31% of world electronics exports just before the pandemic, while it became 18.76% during the pandemic in 2020 and 18.14% in 2021. It is estimated that the size of the world electronics market will increase by 5% to $1.3 trillion by 2025. Meanwhile, the Asia-Pacific region appeared as an important region voicing over about 49.4% of the 2020 global electronics market [41].

**Table 3:** Share of the G-7 countries in world electronics exports (thousand $).

Year	World	G-7	
	Total	Total	Percentage (%)
**2021**	3,304,353,922	599,395,222	18.14
**2020**	2,775,122,623	520,608,355	18.76
**2019**	2,702,465,625	548,839,776	20.31

**Adopted from:** Trademap 2022

**Fig 10:**
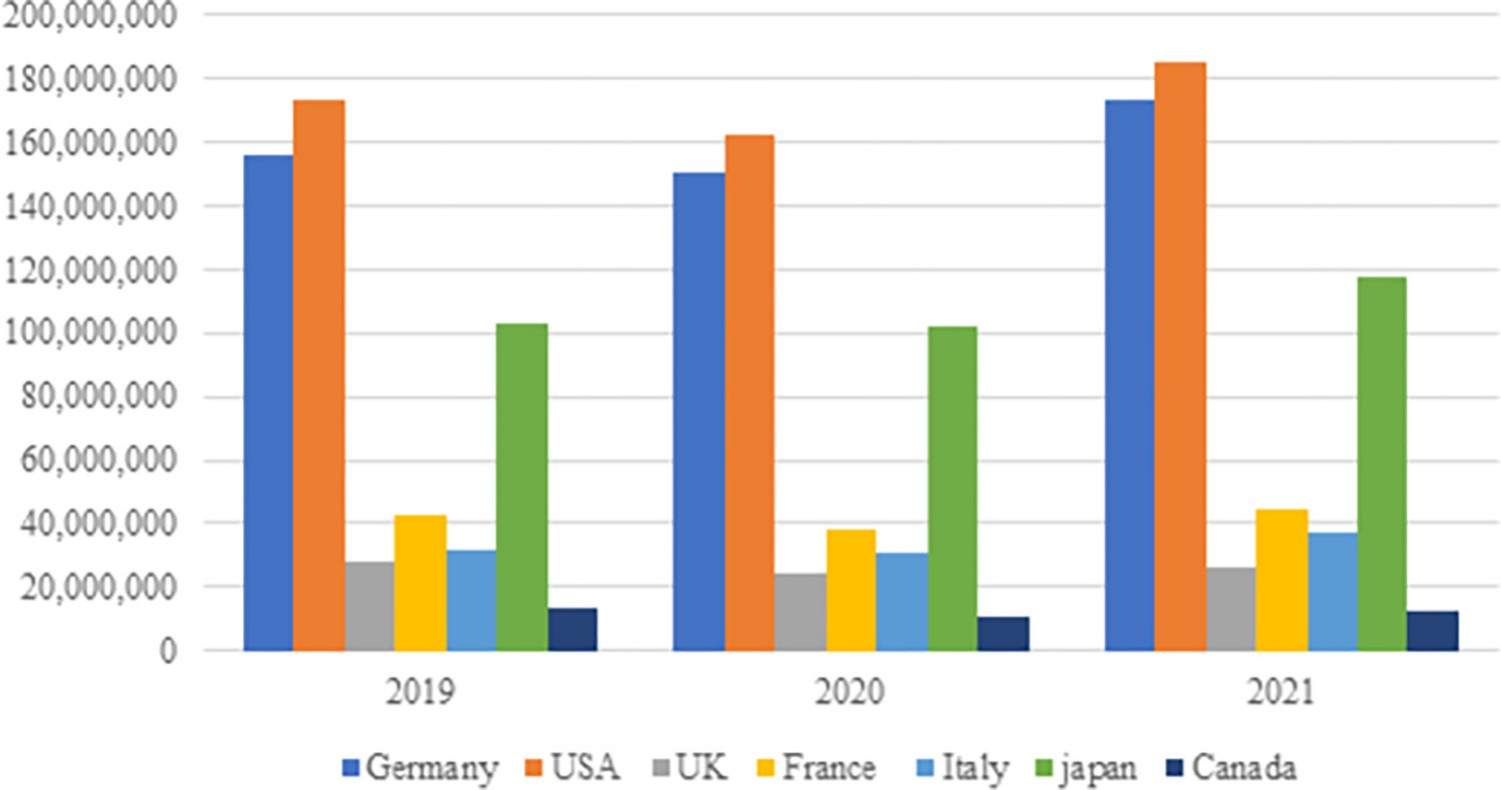
Total electronic product export data of G-7 countries (thousand $). **(Adopted from:** Trademap 2022**)**.

The electronics export data of the G-7 countries are shown in Table 4 and Fig 10. Accordingly, it can be proposed that the pandemic disrupted the electronics exports of these countries in 2020. The two countries witnessing the highest decline in their exports in 2020 compared to 2019 became the USA and Germany. However, there was a significant increase in the electronics exports of the G-7 countries in 2021 thanks to the introduction of novel treatment methods, the lifting of some pandemic-related restrictions, and, therefore, the decrease in the effects of the pandemic.

**Table 4:** Electronics export data for the G-7 countries (thousand $).

Countries / Years	2019	2020	2021
Germany	156,286,307	150,473,979	173,533,163
USA	173,001,470	162,785,011	185,404,509
UK	28,002,935	24,678,960	26,415,271
France	42,924,656	38,203,071	44,976,719
Italy	32,062,523	30,969,754	37,538,018
Japan	103,041,446	102,497,089	117,881,568
Canada	13,520,439	11,000,491	12,603,620

**Adopted from:** Trademap 2022

**Fig 11:**
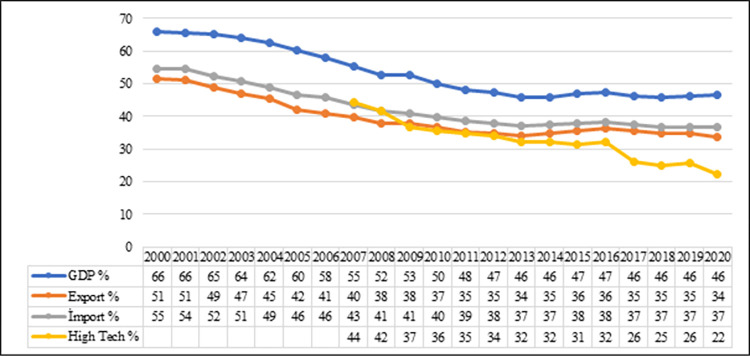
Share of G-7 countries in GDP, exports, imports and high technology exports (%). **(Adopted from:** World Bank 2022**)**.

The World Bank data for 2020 [42] revealed the total GDP of the countries to be $38.7 trillion, their total imports to be $7.6 trillion, and their total exports to be $7.1 trillion. The share of the G-7 countries in world trade was 37% in imports and 34% in exports in 2020. In addition, these countries realize 22% of the world’s high-tech product exports (Fig 11). Although the share of the G-7 countries in world trade has decreased over the years, the relevant data show that G-7 still maintains its position as an influential group for world trade.

## 3. The impacts of Covid-19 on the G-7 countries’ international trade

The COVID-19 pandemic has seriously affected all stages of the economic cycle across the world, from production to consumption. Many countries implemented various restrictions and quarantine practices to reduce the spread of the virus, which brought international social, economic, and commercial relations to a standstill. The increasing number of cases and deaths as of 2020 mandated implementing a couple of measures to protect public health, while the leaders of the G-7 countries highlighted the necessity to support global trade and investments, encourage science, research, and technology cooperation, and act together to resolve the health and economic risks by the pandemic to get through this process with the least damage [43]. Since the present study targets the G-7 countries, we presented and interpreted the number of COVID-19 tests, cases, and deaths to bring an in-depth insight into the factors affecting the electronics trade of these countries in the specified periods.

The data reported by Github [44] demonstrates the numbers of COVID-19 tests in the G-7 countries between March 2020 and December 2021; accordingly, they showed an increasing trend between these dates. Despite an evident decrease significantly in June 2021, the increase in the number of tests persisted until November 2021 and peaked in December 2021. In this process, the months with the highest numbers of tests were determined to be December 2021 with 159,236,577 tests and September 2021 with 117,721,431 tests. Based on the same data, it is evident that the Covid-19 case numbers in G-7 countries started to exhibit a rapid increase as of September 2020, during the period spanning from March 2020 to December 2021. The cases started to be reported in these countries increasingly as of September 2020. Despite slight decreases in some months, the increasing trend reached rather high levels as of November 2020. In this process, the months with the highest numbers of cases were determined to be January 2021 with 9,325,379 cases and December 2021 with 14,065,838 cases.

Another data reported by Github [44] indicates the numbers of deaths due to COVID-19 in the G-7 countries between March 2020 and December 2021. We discovered that the number of deaths in these countries peaked in April 2020, December 2020, and January 2021. The deaths showed a rapid increase, especially between November 2020-January 2021. Despite significant drops in the numbers of deaths between January 2020 and July 2021, the deaths started to follow an increasing trend again as of July 2021. In this process, the months with the highest numbers of fatalities became December 2020 with 148,922 deaths and April 2020 with 132,433 deaths.

**Fig 12:**
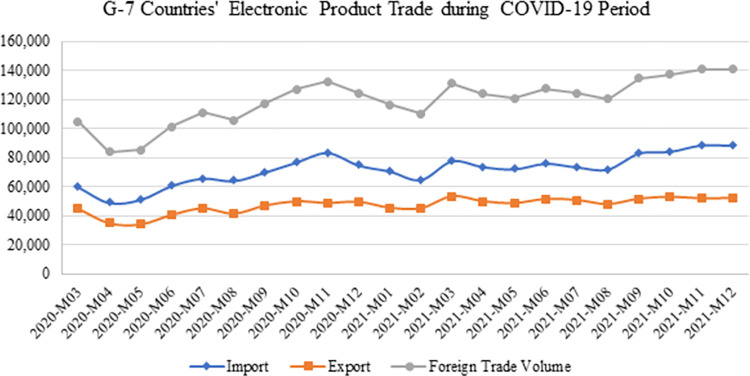
G-7 countries’ electronic product trade during COVID-19 period (thousand $). **(Adopted from:** Trademap 2022**)**.

The monthly total electronic product exports, imports, and trade data for the G-7 countries between March 2020 and December 2021 are presented in Fig 12. During the Covid-19 period, G-7 countries’ electronic product imports showed a general downward trend in April and May 2020, and although there was a downward trend again in January and February 2021, it showed an increasing trend towards the end of the pandemic. Similar to the import trend, the export data also showed a similar trend. It can be argued that fluctuations in electronic product trade were influenced by the pandemic-related restrictions implemented by countries. The quarantine measures imposed by nations have also caused uncertainties in supply and demand. Such measures have also resulted in a decrease in the supply of goods because workers are incapacitated by the health shock, and the closure of port and airport facilities has negatively affected logistics flows, leading to fluctuations in import and export figures [45]. Fig 12 illustrates a decline in electronic product imports and exports during January and the following month of 2021, coinciding with the peak of COVID-19-related fatalities. During this period, countries reimposed restrictions, focusing on reducing the impact of the pandemic instead of increasing trade. However, as mortality rates decreased and testing increased, G-7 nations observed a significant surge in electronic product trade. This could be attributed to factors such as adaptation to distant work environments, online education, and an increase in home entertainment demand [12]. During the Covid-19 period, G-7 countries were able to cover 65% of the total exports with total imports, as shown in Fig 12. G-7 countries focused more on imports during this period. In order to reveal the impact of Covid-19 on the electronic product trade of G-7 countries between March 2020 and December 2021, this study employed panel data analysis in the methodology section to identify the factors influencing the electronic product imports and exports of G-7 countries.

## 4. Literature review

The pandemic caused significant disruptions to global trade, impacting trade flows, volumes, and product categories across various industries and supply chains. The electronic product trade among G-7 countries was particularly affected by the pandemic’s challenges, leading to changes in demand and supply for these products. The effects of the pandemic on electronic products have been substantial. The G-7 countries, namely the United States, Canada, Germany, the United Kingdom, Japan, Italy, and France, have experienced these effects in different ways and to different magnitudes. The shift to distance work and learning in schools due to the pandemic has resulted in a significant increase in demand for electronic devices. This sudden surge in demand has caused disruptions in the supply chain due to the limited supply in response to the high demand. Understanding the trends and patterns in electronic product trade during this period is crucial for policymakers and businesses to make informed decisions and address supply chain disruptions. To the best of our knowledge, although various studies have examined commercial relationships from the past to the present, empirical studies that specifically examine this relationship during the pandemic are limited. This section provides a general overview of existing research on electronic product trade among G-7 countries during the COVID-19 period and attempts to identify trends through these studies.

It is an undisputed fact that the Covid-19 pandemic has had an impact on the electronic product trade of G-7 countries. Countries that had regional trade agreements prior to the pandemic experienced negative effects in bilateral trade flows during the pandemic [6]. According to an OECD [46] report, although the total trade volume during the pandemic period increased considerably compared to the pre-pandemic period, it can be argued that the commercial effects differed across certain goods, services, and trade partners, and that the pandemic put pressure on some sectors and supply chains. The changes in trade flows seen within a single year during the pandemic were comparable to those that could be observed in 4–5 years before the pandemic. The Covid-19 pandemic has significantly affected global trade volume, particularly over the past two years [47]. Restrictions on travel between countries and face-to-face activities during the pandemic have also affected the global supply chain of electronic products [48].

The trade of electronic products within the G-7 countries has been subject to various effects amid the COVID-19 pandemic. As pointed out in [48], supply chain disruptions were mainly determined by the number of countries that imposed restrictions and the duration of quarantine periods during the initial stage of the pandemic. The pandemic has also led to interruptions in global value chains, a shift to distance work systems, and changes in consumer behavior due to restrictions, which have impacted both the supply and demand for electronic products [49]. The impact of economic contractions experienced by countries during the pandemic on global trade has affected the demand for electronic products [47]. Thus, the impact of the pandemic on electronic product trade in G-7 countries is contingent on various factors, including the pandemic’s severity and duration, the effectiveness of measures taken to mitigate its effects, and changes in consumer behavior.

G-7 countries, which include major economies like the USA, Germany, and Japan, play a significant role in global electronic product trade, acting as primary importers and exporters of electronic goods across the world. The Covid-19 pandemic has had an impact on electronic product trade in these countries, leading to several studies that examine its effects through the use of complex network analysis and panel data analysis. Different aspects of electronic product trade, including disruptions in supply chains, changes in demand patterns, and government policies in response to the pandemic, have been investigated by various studies. For example, [8] conducted research on the effects of Covid-19 on specific sectors, including the electronic product sector.

Several macroeconomic studies, such as [7, 9, 50], have investigated the effects of COVID-19 on trade and economic activities. These studies have utilized various techniques to investigate trade relationships between countries and the influence of government policies on trade flows, such as complex network analysis [6, 51]. Additional research, such as [8], has scrutinized specific sectors, including automotive, energy, agriculture, education, travel and tourism, and consumer electronics. For instance, [7] identified significant adverse effects of the pandemic on the electronic industry in India, while [8] revealed significant negative impacts on the aforementioned sectors. Additionally, [9] concluded that the pandemic had significant adverse effects on industrial production in India. Moreover, [50] observed that the effects of the pandemic on food security were more noticeable in low-income countries, but more prominent in upper-middle-income economies. Several studies have explored the effects of COVID-19 on international trade in different contexts. In their study, [6] analyzed changes in bilateral trade flows of 68 countries that exported to 222 destinations during the pandemic and found that government policies had negative effects on trade flows, with the most significant negative impact observed in exports between high-income countries. [51] examined the impact of green coffee beans on the global trade network using social network analysis and concluded that the pandemic significantly altered bilateral trade relations, resulting in a decrease in the total trade volume and a reduction in the number of commercial connections. [52] investigated how the effects of COVID-19 on international trade changed over time and found that the pandemic had a significantly negative impact on international trade. In their study, [53] found that the pandemic disrupted the global value chain of the manufacturing industry, leading to a slowdown in production, reduced efficiency, and increased costs. Lastly, [54] studied the world trade network with a focus on ASEAN countries, but found no evidence of significant changes in centrality after the pandemic for most ASEAN and major trading countries.

Several studies have utilized panel data analysis to understand the impact of Covid-19 on the electronic product trade of G-7 countries. [7] applied this analysis to the Indian economy and found that the pandemic had a significant impact on growth, manufacturing, trade, and the MSME sector. Similarly, [50] investigated the effects of the pandemic on the commercial and economic aspects of food security in 45 developing countries using panel data analysis and found that it caused significant disruptions in the food supply chain.

Regarding the impact of government policies on trade during the Covid-19 period, [6] found that the responses to government policies had a significant impact on trade flows. They suggest that future studies can investigate how successful these policy responses were in mitigating the negative effects of the pandemic on trade flows. Finally, [53] revealed that Covid-19 has had negative effects on the global value chain of the manufacturing industry, resulting in disruptions in the supply chain and a decrease in demand.

Upon reviewing the existing literature, it is evident that the Covid-19 pandemic has had significant adverse effects on global trade flows, particularly in the electronic products industry. The effects of the Covid-19 pandemic on electronic product trade have been attributed to various factors, including disruptions in global supply chains, decreased consumer demand, and changes in government policies and regulations. Complex network analysis and panel data analysis have been utilized in studies to investigate the influence of these factors on trade relationships between countries and potential opportunities for electronic product trade. In general, these studies suggest that the pandemic has had widespread impacts on electronic product trade in G-7 countries. The results of these investigations are anticipated to offer significant guidance for policymakers and industry professionals to create efficient approaches for adjusting to the altering global trade situation and lessening the impacts of the pandemic.

## 5. Methodology

In this study, we addressed electronics exports of the specified countries during the pandemic using two different methods. First, we mapped the intra-group electronics exports of the G-7 countries using the network analysis and determined the hub and authority countries in the network. Then, the variables affecting these exports during the pandemic were examined using panel data analysis. Details of the methods are presented as follows:

### Method-1

Network theory indeed relies on graph theory. In its simplest definition, a network can be visualized as lines connecting two or more points.

- G = (V, E) is the overall graph representation, where:
  ○ V is the vertex set,
  ○ E is the edge set.

The points connecting the lines are called nodes or actors, and a line is called an edge. Ties in networks can be directional or non-directional. In the study, we generated an export network with directional ties. The nodes in the graph, formed by the export network, show the countries, and the trade relations between the countries demonstrate the ties. The distinctive feature of the generated export network is that it is an ego network matrix. Ego-centric networks are usually those from a particular set of actors (egos) and between their mutual ties (alters) [55].

In the analysis, nodes with a high number of ties are called hubs, and nodes with a high number of ties from other nodes are called authorities [56]. Hyperlink Induced Topic Search (HITS) algorithm is utilized when calculating authority and hub nodes.

Networks are divided into weighted and binary networks, and the HITS algorithm calculates hub and authority scores in both networks and informs about the degree of connectivity of nodes. Thus, different from the results of the countries’ trade shares, the findings yield a new ranking for the trade networks by their centrality [57].

The HITS algorithm is implemented for economic networks as follows: Each node *i* in the network is assigned values as (*x*_*i*_) authority and (*y*_*i*_) centrality. A node with high authority refers to its ties from many highly centralized nodes. On the other hand, a node with high centrality refers to that this node has outgoing ties to many nodes with high authority [58].

Authority scores are proportional to the sum of the centralities of the nodes with incoming ties.


$$x_{i}=\sum_{j}A_{ij}y_{j}$$ (1)

The centrality of a node is proportional to the sum of the authorities of the nodes with incoming ties from the node.


$$y_{i}=\beta \sum_{j}A_{ij}x_{j}$$ (2)

Values *a* and *β* constant in the formulas. An *A*_*ij*_ matrix element is included in both equations, and *A*_*ij*_ matrix elements consist of ties defining the centrality of node *i*. Accordingly, the above-mentioned formulas are generated as a matrix as follows [58]:

x=αAy

$$y=\beta A^{T}x$$

specified above can be denoted as:

$$AA^{T}x=\lambda x$$

$$A^{T}Ay=\lambda y$$

*λ* takes (*aβ*)^−1^; thus, authority and hub centrality scores are calculated with *AA*^*T*^ and *A*^*T*^*A* eigenvalues.

### Method-2

In the study, we compared the years 2019, 2020, and 2021 to discuss the cases during the pandemic and the pre-pandemic periods separately. For this purpose, we utilized the data, extracted from the Trademap database, on electronics exports between the G-7 countries in the specified years. In network analysis, it is rather important to reach all nodes in the group to be able to understand the emerging network.

The data utilized in the study were structured in 8 x 8 matrices for each G-7 country’s and China’s electronics exports with the others for the years 2019, 2020, and 2021; thus, we were able to demonstrate the export relations of the countries collectively. The created three matrices for the specified years were analyzed using the UCINET program, and the findings were discussed to portray the trade relations between the countries. In addition to network analysis, we also addressed the impacts of the pandemic on the intra-group electronics trade using the panel data analysis method (econometric analysis). Accordingly, we generated two models, imports and exports, where 22 months of data were used (from March 2020 to December 2021) based on the WHO’s declaration of COVID-19 as a pandemic on March 11, 2020 [59]. To test the impacts of the pandemic on the electronics exports of the G-7 countries, we adopted population, number of COVID-19 tests, number of cases, number of deaths, positivity rate, the ratio of the number of tests to the population, bed occupancy rate, and the ratio of the number of deaths to the number of cases as independent variables, while using imports and exports data of the countries as dependent variables. The variables in the models are explained below.

**Import**_**ijt**_: is the import of country i from country j in period t.

**Export**_**ijt**_: is the export of country i from country j in period t.

**Population**_**it**_: is the population of country i in period t.

**Number of tests**_**it**_: is the number of COVID-19 tests in country i in period t.

**Number of cases**_**it**_: is the number of COVID-19 cases in country i in period t.

**Number of deaths**_**it**_: is the number of people who died due to COVID-19 in country i in period t.

**Positivity rate**_**it**_: is the ratio of people testing positive for COVID-19 to the number of tests per day in country i in period t.

**Number of tests/population**_**it**_: is the ratio of the number of COVID-19 tests to the population in country i in period t.

**Bed Occupancy Rate:** is the ratio of inpatients with COVID-19 to the total number of beds in hospitals in country i in period t.

**Number of deaths**_**it**_**/cases**_**it**_: is the ratio of the number of people who died due to COVID-19 to the number of cases in country i in period t.

Below are the models built using the variables mentioned above.

lnimport_ijt_ = β_0_ + β_1_population_it_ +β_2_lnnumberoftests_it_ + β_3_numberofcases_it_ + β_4_numberofdeaths_it_ + β_5_lnpositivityrate_it_ + β_6_lntest_population_it_ + β_7_lnoccupancyrate_it_ + β_8_lndeath_case_it_ + ε_it_

lnexport_ijt_ = β_0_ + β_1_population_it_ +β_2_lnnumberoftests_it_ + β_3_numberofcases_it_ + β_4_numberofdeaths_it_ + β_5_lnpositivityrate_it_ + β_6_lntest_population_it_ + β_7_lnoccupancyrate_it_ + β_8_lndeath_case_it_ + ε_it_ While β_0_ represents the constants in the models, β_1_, β_2_, β_3_, β_4_, β_5,_ β_6,_ β_7,_ and β_8,_ preceding the variables are the coefficients to be predicted. ε_it_ is the error term in the models.

While i represents the country addressed, t represents time.

The list of the G-7 countries addressed in the analysis is shown in Table 5.

**Table 5:** G-7 countries in the study.

Germany
USA
UK
France
Italy
Japan
Canada

Econometric analyses often utilize three data types: time series, cross-sectional data, and panel data [60]. What differs panel data from time series and cross-sectional data is that panel data bears both horizontal and vertical dimensions [61]. Comparing panel data models with horizontal or vertical time series data models, panel data have become more common in analyzing increasingly complex human behaviors in scholarly work.

In case individual behaviors are identical depending on specific variables, panel data consider diverse variables in addition to these behaviors and offer a chance to learn about one’s behaviors. Thus, one’s behaviors can be interpreted more accurately with the help of more and more diverse data [62]. In this context, we can propose that panel data analysis is utilized since only cross-sectional data may provide a poor explanation of the models in econometric research. In addition to more data and variability, panel data ensure less linearity between the variables and more efficient findings with the generated models [63].

## 6. Findings

### Complex network analysis of intra-group electronics exports of the G-7 countries and China

**Fig 13:**
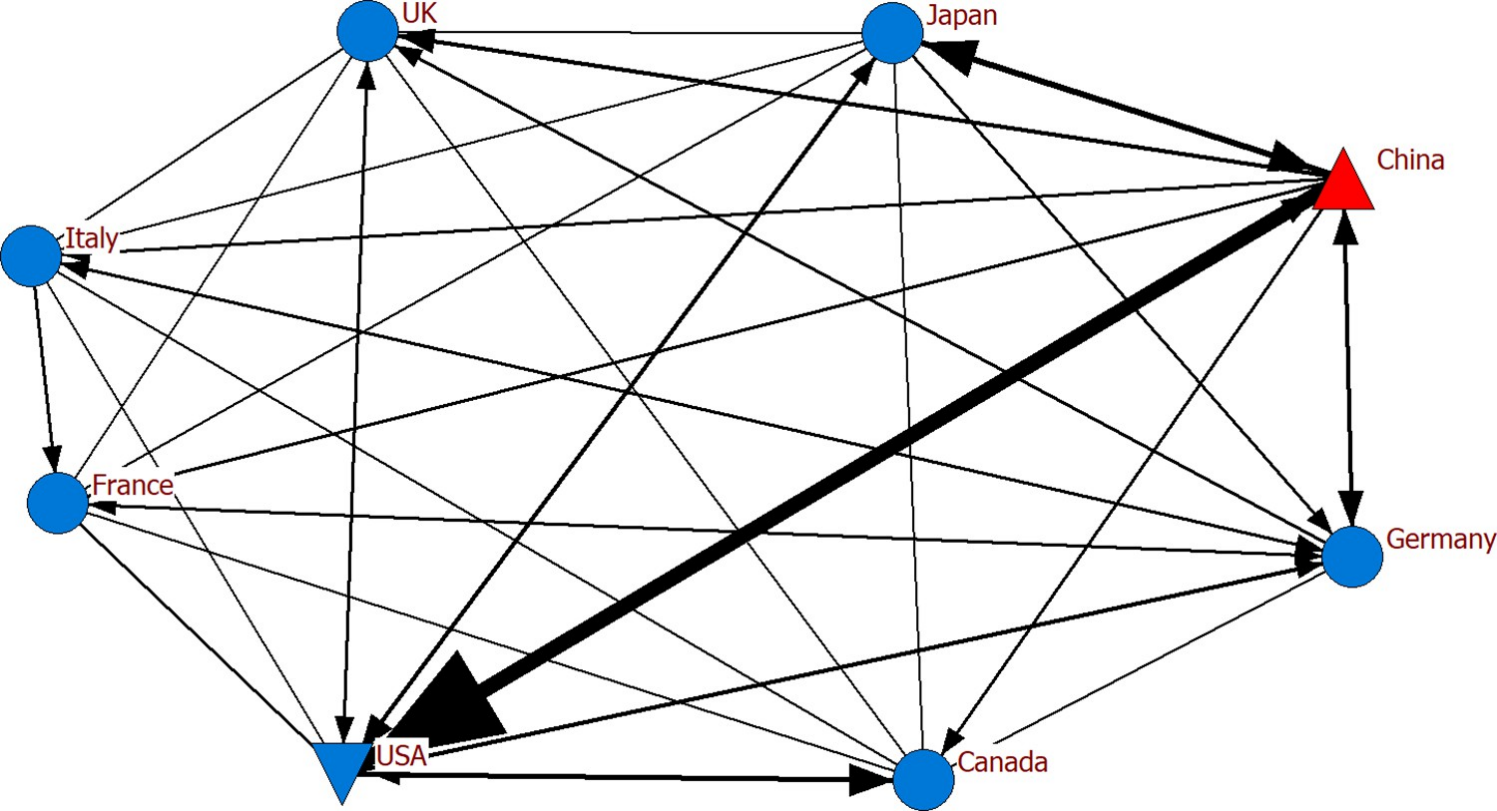
Export network of G-7 countries and China for 2019.

**Fig 14:**
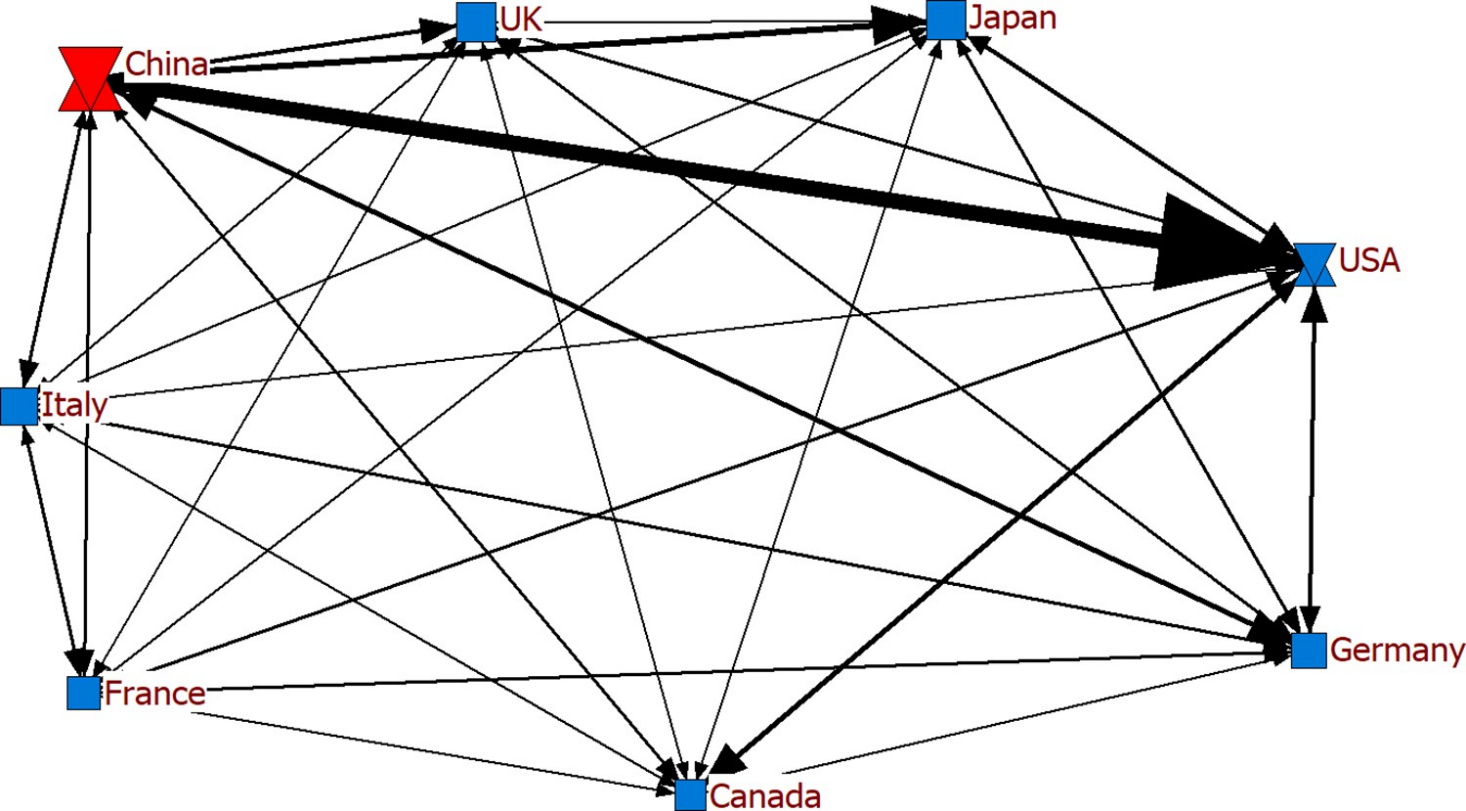
Export network of G-7 countries and China for 2020.

**Fig 15:**
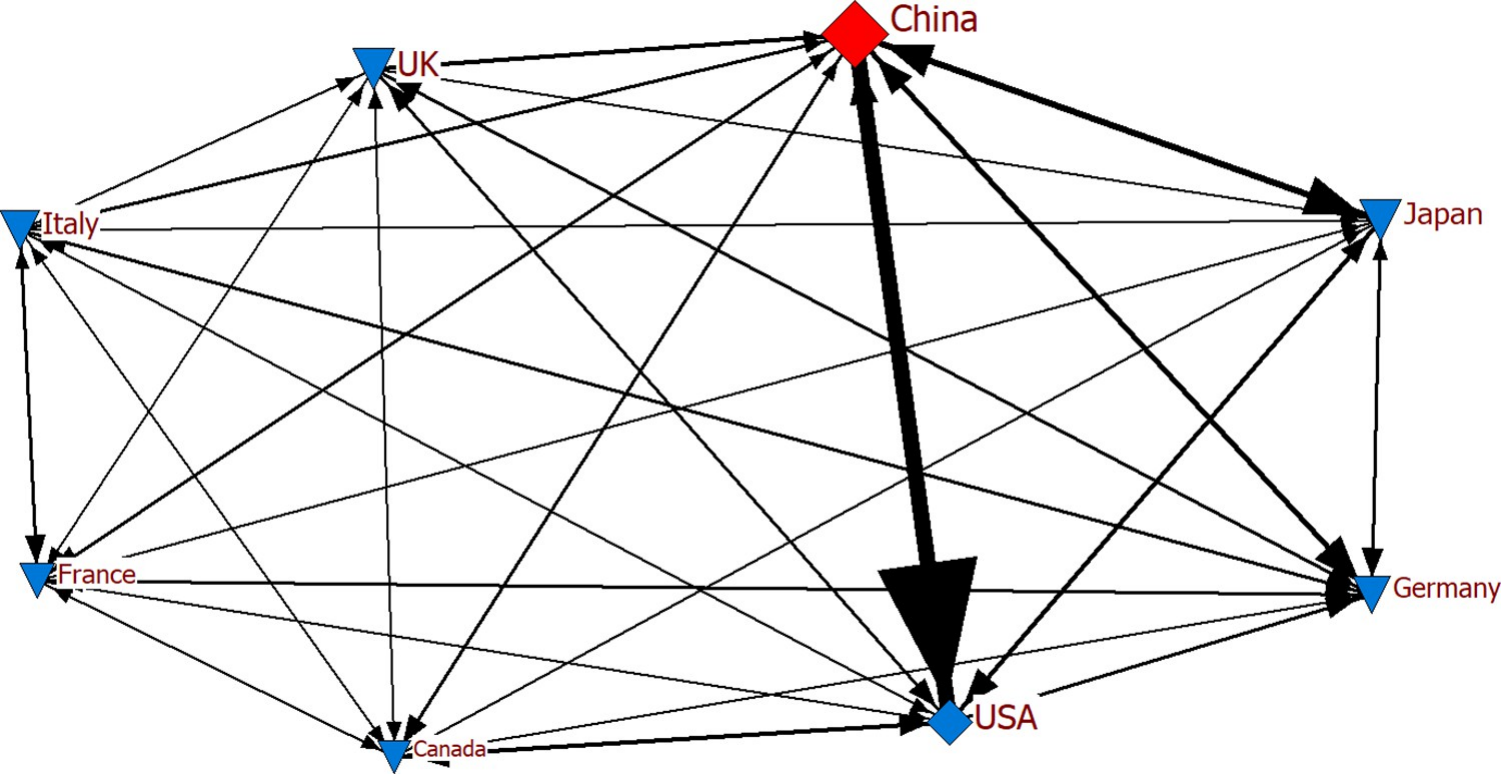
Export network of G-7 countries and China for 2021.

In the analysis, we initially detected the hub and authority countries. According to Table 6, China was the hub country in exports among G-7 countries and China in the specified years, while the country with the most ties became the USA for 2019, 2020 and 2021. Considering the networks where China appeared as ego (Figs 13–15), the findings revealed its frequent and intense exports to the USA. The thickness and frequency of the tie may document the intensity of the trade relations between the two countries. It can be asserted the intensity of the trade relations may be because the two countries are almost neighbors. Despite its trade with all G-7 countries, we determined that the other countries with which China had the most ties in electronics exports, apart from the USA, were United Kingdom, Japan and Germany in these three years. With the availability of the COVID-19 vaccine and the lifting of restrictions and quarantine measures as of 2021, the production volume increased compared to 2020, positively affecting economic activities and trade relations between countries. Given the network structure of 2021, Germany also had increased commercial relations compared to 2020.

**Table 6:** Hub and authority statistics of the G-7 countries and China for 2020.

2019			2020			2021		
Countries	Hub	Authority	Countries	Hub	Authority	Countries	Hub	Authority
Germany	0.125	0.166	Germany	0.113	0.175	Germany	0.109	0.191
**USA**	0.047	**0.930**	**USA**	0.041	**0.931**	**USA**	0.037	**0.930**
UK	0.038	0.123	UK	0.033	0.127	UK	0.030	0.122
France	0.038	0.069	France	0.032	0.064	France	0.028	0.068
Italy	0.025	0.067	Italy	0.026	0.064	Italy	0.027	0.073
Japan	0.137	0.274	Japan	0.123	0.264	Japan	0.121	0.258
Canada	0.081	0.070	Canada	0.063	0.070	Canada	0.059	0.069
**China**	**0.976**	0.053	**China**	**0.982**	0.050	**China**	**0.983**	0.046

Table 7 shows ego network sizes, number of ties, number of pairs, and densities of intra-group electronics exports of the G-7 countries and China in 2019, 2020, and 2021. Given the basic statistics in the network of the specified years, the number of ego networks was 7 for each country in clusters where actors were considered egos, and the number of ties for each country was 42. Since all countries export with each other, ego network sizes of all networks are expected to be the same. The density coefficient is denoted by the ratio of the number of existing ties in the network to the highest possible number of ties [64] and is equal to 1 for all countries. Accordingly, all countries had mutual exports and robust communication and information exchange within the network.

**Table 7:** Key statistics on electronics exports of the G-7 countries and China in 2019, 2020, and 2021.

2019-2020-2021				
Countries	Size	Ties	Pairs	Density
Germany	7	42	42	100
USA	7	42	42	100
UK	7	42	42	100
France	7	42	42	100
Italy	7	42	42	100
Japan	7	42	42	100
Canada	7	42	42	100
China	7	42	42	100

The centrality measures of the clusters are given in Tables 8 and 9. The eigenvector centrality measure (Table 8) is a parameter measured high if an actor has a neighborhood relationship with actors with high ties [56]. Eigenvector centrality is one of the methods used to determine the importance of a node in a graph. This method is based on the principle that a connection to an important node is more valuable than a connection to a less important one. In other words, this centrality measure indicates that a node’s centrality increases more with its connection to a large number of connected nodes than with its connection to relatively fewer connected nodes [65].


$$\sigma_{E}(i)=\frac{1}{\lambda_{max}(A)}\sum_{i=1}^{n}{a_{ji}v}_{j}$$

**Table 8:** Eigenvector centrality statistics of the electronics exports network of the G-7 and China.

2019		2020		2021	
Countries	Eigenvector	Countries	Eigenvector	Countries	Eigenvector
Germany	0.182	Germany	0.182	Germany	0.191
USA	0.645	USA	0.646	USA	0.645
UK	0.108	UK	0.107	UK	0.100
France	0.068	France	0.062	France	0.064
Italy	0.060	Italy	0.059	Italy	0.067
Japan	0.254	Japan	0.242	Japan	0.238
Canada	0.166	Canada	0.149	Canada	0.141
China	0.663	China	0.670	China	0.672

**Table 9:** Degree centrality statistics of the electronics export network of the G-7 countries and China.

Years	2019				2020				2021			
Countries	Outdeg	Indeg	nOutdeg	nIndeg	Outdeg	Indeg	nOutdeg	nIndeg	Outdeg	Indeg	nOutdeg	nIndeg
Germany	55796984	43198848	0.075	0.058	54338188	42983772	0.070	0.055	63035692	54650296	0.067	0.058
USA	57683384	151146448	0.078	0.203	54859156	152712496	0.070	0.195	60878480	182223248	0.064	0.193
UK	11633112	29855364	0.016	0.040	10339689	28495410	0.013	0.036	11377268	31895260	0.012	0.034
France	16993222	24839882	0.023	0.033	14664387	22697662	0.019	0.029	16686511	27440440	0.018	0.029
Italy	11625585	19365084	0.016	0.026	11635059	18503200	0.015	0.024	14569135	24047214	0.015	0.025
Japan	45806544	39944732	0.062	0.054	45338612	39529248	0.058	0.051	53454076	45469328	0.057	0.048
Canada	11161666	34188192	0.015	0.046	9023788	31383300	0.012	0.040	10339625	35710368	0.011	0.038
China	190283952	58445904	0.256	0.079	200448528	64342300	0.257	0.082	245162944	74067584	0.260	0.078

The vector υ = (υ_1_, …, υ_n_)^T^ in the formula represents the eigenvector of the adjacency matrix A corresponding to its largest eigenvalue *λ*_max_(A) [58].

Eigenvector calculations are performed on the adjacency matrix for eigenvector centrality. As shown in Table 8, the two countries with the highest eigenvector centrality for the G-7 and China in the specified years were found to be China and the USA. These countries are known to be relatively strong in terms of electronic product exports. In the study, we obtained similar findings considering the countries with the highest centrality and number of ties (Table 6, Figs 13–15). The volume of trade between geographically close countries is remarkable [66]. A relatively intense geographical trade is generally attributed to geographical proximity [67, 68]. The fact that Canada and the USA are also regionally close has an impact on their prominence in the network.

Degree centrality is a measure of the interconnectedness of nodes in a network. The degree of a vertex in a network is the number of edges connected to it. In mathematical terms, the degree k_i_ of a vertex i is k_i_ = n.

$$k_{i}=\sum_{j=1}^{n}A_{İJ}$$

The centrality of node i increases proportionally with the degree centrality value [72]. In other words, the higher this value is, the higher the centrality of node i.

Table 9 shows the ranks of these countries by their degree centrality in 2019, 2020, and 2021. Out-degree in this table is a parameter representing the number of countries to which a country exports, while the in-degree parameter represents the number of countries exporting with that country [69]. Accordingly, we determined that China had the most export ties in these networks and was the most active actor of the network by its degree centrality as the country to which the most exports were directed in the years considered. In this analysis, China was followed by the USA.

### Panel data analysis of electronic product exports among G-7 countries

In order to evaluate the relationships between the dependent and independent variables, a correlation matrix was constructed as depicted in Table 10. The matrix revealed that export (dependent variable) had a negative relationship with the variables of bed occupancy rate, number of tests/population, and number of deaths/number of cases ratio, while it had a positive relationship with all other variables. On the other hand, import (the other dependent variable) was negatively associated with the variable of number of deaths/number of cases and positively associated with all other independent variables.

**Table 10:** Correlation matrix.

Correlation	LOGEXPORT	LOGIMPORT	LOGPOPULATION	LOGNUMBEROFTESTS	LOGNUMBEROFCASES	LOGNUMBEROFDEATHS	POSITIVITYRATE	BEDOCCUPANCYRATE	TESTS_POPULATION	DEATHS_CASES
LOGEXPORT	1									
LOGIMPORT	0.6926	1								
LOGPOPULATION	0.7568	0.5958	1							
LOGNUMBEROFTESTS	0.2314	0.6230	0.3326	1						
LOGNUMBEROFCASES	0.3179	0.6209	0.4033	0.8445	1					
LOGNUMBEROFDEATHS	0.2655	0.5509	0.4460	0.6192	0.8079	1				
POSITIVITYRATE	0.1358	0.1588	0.1256	0.0066	0.3178	0.3581	1			
BEDOCCUPANCYRATE	-0.2584	0.1110	-0.3057	0.2364	0.2359	0.4734	0.1950	1		
TESTS_POPULATION	-0.2300	0.1049	-0.1078	0.6643	0.4804	0.1951	-0.1146	0.0451	1	
DEATHS_CASES	-0.2570	-0.2778	-0.0643	-0.3903	-0.3385	0.1618	0.0373	0.2908	-0.3043	1

In order to assess the potential for multicollinearity among the variables, a Variance Inflation Factor (VIF) test was employed. The presence of multicollinearity is indicated by a VIF value greater than 10, while a value less than 10 suggests no multicollinearity [70]. In this case, the variables Lognumberofcases (15.11961) and Lognumberofdeaths (12.14759) were found to exhibit multicollinearity. To address this issue, the first differences of the variables were calculated, and multicollinearity was resolved. Following these adjustments, the VIF values were less than 10, and the test results are presented in Table 11.

**Table 11:** VIF test results.

Variable	Coefficient	VIF Value
	Variance	
LOGPOPULATION	0.061426	3.065384
LOGNUMBEROFTESTS	0.029087	6.087110
D LOGNUMBEROFCASES	0.019741	1.951733
DLOGNUMBEROFDEATHS	0.017282	1.888922
POSITIVITYRATE	3.200693	1.310425
TESTS_POPULATION	2.062839	3.498785
DEATHS_CASES	11.35818	2.066189
BEDOCCUPANCYRATE	0.000125	2.518751
C	11.16968	NA

According to Baltagi [71], it is unnecessary to perform a unit root test when the time dimension of the data is between 25 and 250. In the panel data analysis following complex network analysis, we used 22 months of data to examine the impacts of COVID-19 on the electronics trade of the G-7 countries. Accordingly, since the time dimension of the data did not correspond to 25–250, we could not perform the unit root test and had to launch the panel data analysis stage directly.

In the model selection process, some preliminary tests were conducted separately for the import and export dependent variables. The unit effects of the models were analyzed using the F and Breusch Pagan tests. The results of these analyses are presented in Table 12.

**Table 12:** Breusch-Pagan and F test results.

Test Type	Model I (Export)		Model II (Import)	
	Chi-Square Test Statistics	P Value	Chi-Square Test Statistics	P Value
F Test	144.448910	0.0000	217.558619	0.0000
Breusch-Pagan Test	853.8724	0.0000	546.4784	0.0000

Initially, the null hypothesis of "the variance of the unit effect is equal to zero" was rejected based on the F test conducted on fixed effects. Later, the null hypothesis was also rejected in the Breusch-Pagan test conducted on random effects using generalized least squares. These test results confirm the presence of a unit effect. Due to the presence of a unit effect, the Pooled Ordinary Least Squares (POLS) method is not appropriate. In this study, we performed the Hausman test to decide which of the fixed or random effects models would be appropriate in the regression model created for panel data analysis.

The hypotheses set for the *Hausman* test are as follows:

H_0_: The random effects model is favored.
H_1_: The fixed effects model is favored.

The result of the *Hausman* test yielded a probability of greater than 0.05 (0.0703 and 0.1194) for both models (Table 13). Therefore, we accepted H_0_ where the random effects model is favored and rejected H_1_ where the fixed effects model is favored.

**Table 13:** Results of the Hausman test.

Model	*χ* ^*2*^	*p-value*	Random/fixed
Model 1 (Export)	14.469548	0.0703	Random
Model 2 (Import)	12.786223	0.1194	Random

Heteroskedasticity, autocorrelation and cross-sectional correlation problems are frequently encountered in panel data analysis, and some tests are available to check these problems. In the study, tests appropriate for the random effects model were applied to identify deviations from the underlying assumptions of the model in the estimated results. Following the Hausman test, the probability value of the Heteroskedasticity LR test was sought to determine whether there was an ununiform varying variance in the study. Accordingly, the relevant hypotheses are given below.

H_0_: There is not heteroskedasticity.
H_1_: There is heteroskedasticity.

p values in the LR test were found to be less than 0.05 for both import and export models in Table 14. According to these values, the null hypothesis H_0_ stating that there is no heteroskedasticity has been rejected, and the alternative hypothesis H_1_ indicating the presence of heteroskedasticity has been accepted.

**Table 14:** Heteroskedasticity, autocorrelation and cross-sectional correlation test results.

	Model I (exports)		Model II (imports)	
Test Type	Test Statistics	p-value	Test Statistics	p-value
**Heteroskedasticity Test**				
Heteroskedasticity LR Test	206.5038	0.0000	74.81061	0.0000
**Autocorrelation Test**				
Wooldridge Test	14.436	0.0090	43.255	0.0006
**Cross-sectional Correlation Test**				
Pesaran CD Test	4.816537	0.0000	7.289855	0.0000

On the other hand, we utilized the Wooldridge test to reveal any autocorrelation problem in the study. In the case of a *p*-value < 0.05 in the test, the H_0_ is rejected, and it is accepted that the error terms have an autocorrelation. Based on the probability value being less than 0.05 in Table 14, the alternative hypothesis H_1_ is accepted, and the presence of autocorrelation problem is detected.

The Pesaran CD test was used to test for cross-sectional correlation, and the null hypothesis H_0_ stating that there is no cross-sectional correlation was rejected due to the probability value being less than 0.05 in Table 14. Therefore, it was concluded that there is cross-sectional correlation according to the random effects model. The analyses conducted revealed that there are problems with heteroskedasticity, autocorrelation, and cross-sectional correlation according to the random effects model.

According to the results of the LR test, we applied White’s cross-section covariance coefficients method to eliminate the ununiform variance problem in both import and export models. In addition, in the case of ununiform variance and/or autocorrelation problems, the estimated generalized least squares method (EGLS) was utilized in both models to achieve more accurate results [72].

Table 15 presents the results of the least squares (LS)-predicted panel regression analysis of the import and export models. In Table 15, White’s cross-section covariance coefficients method and the GLS method were applied in the regression analysis to eliminate the problem of autocorrelation and ununiform variance in the models. Upon relevant corrections, the results of the panel EGLS-predicted regression analysis are given in Table 16.

**Table 15:** Results of the LS-predicted panel regression analysis.

Independent Variables	Models			
	Model I (exports)		Model II (imports)	
	Coefficient	*p*	Coefficient	*p*
CONSTANT	2.769552***	0.0092	5.378117***	0.0000
LOGPOPULATION	0.594125***	0.0000	0.216406***	0.0010
LOGNUMBEROFTESTS	0.054849	0.3073	0.304867***	0.0000
LOGNUMBEROFCASES	-0.070536	0.1120	0.018466	0.6121
LOGNUMBEROFDEATHS	0.009759	0.8134	-0.017781	0.6018
POSITIVITYRATE	0.390499	0.4879	0.356682	0.4420
TESTS_POPULATION	-1.579261***	0.0006	-1.763683***	0.0000
BEDOCCUPANCYRATE	-0.000147	0.9666	0.003921	0.1774
DEATHS_CASES	-4.477193***	0.0000	-0.967442	0.2689
Hausman (Chi-Square)	14.469548		12.786223	
Hausman *p* (Chi-Square)	0.0703		0.1194	
Model	Random Effects		Random Effects	
Adjusted *R*^*2*^	0.657318		0.680724	
Durbin Watson	0.243172		0.313199	
Wooldridge *p*	0.0090		0.0006	
*F*	35.76661		39.64408	
*p* (*F*-statistic)	0.000000		0.000000	
Panel cross-section Heteroskedasticity LR Test (*p*)	0.0000		0.0000	

*** Significant at 1%

** Significant at 5%

* Significant at 10%

**Table 16:** Results of the panel EGLS-predicted panel regression analysis (cross-section weighted).

Independent Variables	Models			
	Model I (exports)		Model II (imports)	
	Coefficient	*p*	Coefficient	*p*
CONSTANT	2.769552***	0.0041	5.378117***	0.0000
LOGPOPULATION	0.594125***	0.0000	0.216406***	0.0027
LOGNUMBEROFTESTS	0.054849	0.2972	0.304867***	0.0000
LOGNUMBEROFCASES	-0.070536*	0.0827	0.018466	0.6199
LOGNUMBEROFDEATHS	0.009759	0.7942	-0.017781	0.6475
POSITIVITYRATE	0.390499	0.6303	0.356682	0.3074
TESTS_POPULATION	-1.579261***	0.0033	-1.763683***	0.0000
DEATHS_CASES	-4.477193***	0.0001	-0.967442	0.1683
BEDOCCUPANCYRATE	-0.000147	0.9718	0.003921	0.1461
Hausman (Chi-Square)	14.469548		12.786223	
Hausman *p* (Chi-Square)	0.0703		0.1194	
Model	Random Effects		Random Effects	
Adjusted *R*^*2*^	0.65731		0.680724	
Durbin Watson	0.243172		0.313199	
*F*	35.76661		39.64408	
*p* (*F*-statistic)	0.000000		0.000000	

*** Significant at 1%

** Significant at 5%

* Significant at 10%

An adjusted *R*^*2*^ value refers to how much of the changes in dependent variables are explained by independent variables. In this study, we calculated adjusted *R*^*2*^ values to be 0.657318 and 0.680724 for Model I and Model II, respectively. In other words, 65% of the imports were explained by the specified independent variables in Model I, while the same variables explained 68% of the imports in Model II. Thus, we can propose that the changes in the import and export figures of the G-7 countries in the period covered in the study can be explained by different variables other than the pandemic-related variables. However, since we investigated the impacts of the pandemic on the electronics trade of the G-7 countries, other variables expected to affect imports and exports could not be included in the analysis.

The results of the Panel EGLS analysis (Table 16) showed that the independent variables affecting the intra-group electronic product trade of the G-7 countries had different effects in both models. In Model I, the deaths/cases ratio, the tests/population ratio and the number of cases significantly and negatively affected exports (*p* < 0.01 and < 0.10, respectively), while the population significantly and positively affected exports (*p* < 0.01). On the other hand, in Model II, the tests/population ratio significantly and negatively affected imports (*p* < 0.01), while the population and the number of tests significantly and positively affected imports (*p* < 0.01). Other variables did not have a statistically significant effect in either model.

## 7. Conclusion

The electronic product industry proliferates thanks to technological advancements and increases its share in international trade. Countries desiring to introduce products with high added value and, thus, increase their share in global trade boost their investments in R&D and attempt to keep up with technological advancements to reinforce their position in the electronics industry. Therefore, this industry is also of great importance for the world’s largest economies. Within the G-7 countries, the leading ones in the world electronics industry are known to be the USA, Germany, and Japan. Yet, the pandemic in 2020 brought adverse impacts on the electronics exports of these countries. In 2020, the two countries with the greatest decline in electronics exports compared to 2019 became the USA and Germany. In 2021, the discovery of new treatment methods against COVID-19, the lifting of some restrictions, and, therefore, fading effects of the pandemic brought the revival of electronics exports within the G-7 countries.

In this study, we initially investigated the trade networks within the G-7 countries and China and found prominent countries in the network to be China and the USA. The pandemic has caused production and supply chain disruptions in many countries, resulting in a well-established and strong supply chain for electronic products in China, and many countries have become more reliant on China to ensure the supply of electronic products during this period. The USA is one of these countries. Furthermore, the new normal conditions, such as distance work and learning, have led to an increase in demand for electronic products. Many people, especially during the pandemic, spent more time at home and needed more computers, tablets, smartphones, and other electronic devices. This has resulted in an increase in electronic product trade between the two populous countries, China and the USA. The findings also showed that the countries with which China had the most ties in electronics exports, other than the USA, were United Kingdom, Japan and Germany. Moreover, Germany was discovered to be the most active country in the network, following China and the USA, regarding its export ties in the network.

We also explored the world electronic product trade in terms of exports, imports, and expected growth in the industry. We addressed electronics exports between the G-7 countries and China comparatively for the years 2019, 2020, and 2021—the eyes of the adverse impacts of the pandemic—and uncovered the status of electronics exports based on the network structures in these years. The network analysis suggested which countries led the electronics exports within the group, which countries had solid or weak trade ties, and which countries may be more influential in the exports in the future.

The findings of panel data analysis were also considered to suggest the effects of the pandemic on the mentioned electronics trade in more detail. In this analysis, generated two models using the data of imports, exports, population, number of COVID-19 tests, number of cases, number of deaths, positivity rate, the ratio of the number of tests to the population, bed occupancy rate, and the ratio of the number of deaths to the number of cases between March 2020 and December 2021. According to the findings, the variables affecting the electronic product trade of the G-7 countries yielded different effects in the models. In Model I (exports), we concluded that the deaths/cases ratio, the tests/cases ratio and the number of cases adversely affected exports, while the population positively affected it. Therefore, it can be asserted that the high number of tests, cases and deaths and the restrictions adversely affected business life in the G-7 countries and led to a reduction in electronics exports in the given years. The high population in G-7 countries and the greater number of workforces continuing to work in electronic product exports than other countries has led to an increase in the quantity of electronic products produced, thus increasing electronic product exports during the pandemic period. In Model II (imports), we determined that the tests/population ratio adversely affected the intra-group electronics imports, while the population and the number of tests brought positive effects on the imports. The high ratio of tests/population in G-7 countries, which led to an increase in the number of cases, is believed to have had a reducing effect on electronic product imports due to social and economic mobility restrictions. It can be proposed that the increase in the number of tests, in turn, had an increasing effect on imports thanks to the increased dependence on technology in the conduct of both social and economic relations, doubling the demand for electronic products. Since the G-7 countries are considered the seven largest economies of the world, it was an expected finding that the variable “population” contributed to electronics imports.

Overall, the findings from the analysis of the intra-group electronics trade of the G-7 countries are thought to contribute to the relevant literature. In addition, our findings would guide prospective researchers interested in the subject. As well as the G-7 countries’ intra-group electronics exports, further studies may address the electronics exports of the G-7 countries within world trade, considering the network of which each member country is an ego. For panel data analysis, future research may employ different variables other than COVID-19-related variables and scrutinize electronics exports of these countries to different economic groups. Finally, prospective researchers may generate more diverse networks by considering different countries or country groups and perform more compressing econometric analyses over a broader period of time in the coming years.
